# Investigating effect of mutation on structure and function of G6PD enzyme: a comparative molecular dynamics simulation study

**DOI:** 10.7717/peerj.12984

**Published:** 2022-03-29

**Authors:** Sadaf Rani, Fouzia Perveen Malik, Jamshed Anwar, Rehan Zafar Paracha

**Affiliations:** 1 School of Interdisciplinary Engineering and Sciences (SINES), National University of Sciences and Technology, Islamabad, Federal, Pakistan; 2 Department of Chemistry, Lancaster University, UK, Lancaster, United Kingdom, UK

**Keywords:** G6PD mutants, MD simulations, Binding site, Structural stability, Binding free energy, Catalysis

## Abstract

Several natural mutants of the human G6PD enzyme exist and have been reported. Because the enzymatic activities of many mutants are different from that of the wildtype, the genetic polymorphism of G6PD plays an important role in the synthesis of nucleic acids via ribulose-5-phosphate and formation of reduced NADP in response to oxidative stress. G6PD mutations leading to its deficiency result in the neonatal jaundice and acute hemolytic anemia in human. Herein, we demonstrate the molecular dynamics simulations of the wildtype G6PD and its three mutants to monitor the effect of mutations on dynamics and stability of the protein. These mutants are Chatham (A335T), Nashville (R393H), Alhambra (V394L), among which R393H and V394L lie closer to binding site of structural NADP^+^. MD analysis including RMSD, RMSF and protein secondary structure revealed that decrease in the stability of mutants is key factor for loss of their activity. The results demonstrated that mutations in the G6PD sequence resulted in altered structural stability and hence functional changes in enzymes. Also, the binding site, of structural NADP^+^, which is far away from the catalytic site plays an important role in protein stability and folding. Mutation at this site causes changes in structural stability and hence functional deviations in enzyme structure reflecting the importance of structural NADP^+^ binding site. The calculation of binding free energy by post processing end state method of Molecular Mechanics Poisson Boltzmann SurfaceArea (MM-PBSA) has inferred that ligand binding in wildtype is favorable as compared to mutants which represent destabilised protein structure due to mutation that in turn may hinder the normal physiological function. Exploring individual components of free energy revealed that the van der Waals energy component representing non-polar/hydrophobic energy contribution act as a dominant factor in case of ligand binding. Our study also provides an insight in identifying the key inhibitory site in G6PD and its mutants which can be exploited to use them as a target for developing new inhibitors in rational drug design.

## Introduction

Human Glucose-6-Phosphate Dehydrogenase (G6PD) is a rate-limiting enzyme of the pentose phosphate pathway (PPP) involved in the formation of 6-phosphogluconolactonere leasing NADPH which directs the formation of ribulose 5- phosphate producing nucleotides at the end of the pathway. The PPP is the only source of NADPH in red blood cells (RBC’s) required to protect the cells from oxidative damage caused by reactive oxygen species (ROS) (WHO Working Group, 1989). NADPH maintains the level of reduced glutathione which is vital for the reduction of H_2_O_2_ and oxygen free radicals, thus controlling the concentration of RBC’s proteins including hemoglobin (Desnick et al., 2001). An elevated level of reactive oxygen species causes loss of membrane integrity leading to hemolysis in RBCs (Dessi et al., 1984; Rao, Kottapally & Shinozuka, 1984; Townsend & Tew, 2003).

The *G6PD* gene is located on the x- chromosome consisting of 13 exons and 12 introns (Persico et al., 1986), and the product of this gene is a protein consisting of 515 amino acids with a molecular weight of 59 kDa located in the cytosol (Rattazzi, 1968). Mutations in the *G6PD* gene result in an x-linked hereditary disease known as G6PD deficiency, which is associated with the protein variants having different levels of enzyme activity leading to a wide variety of biochemical and clinical phenotypes (Desnick et al., 2001). G6PD deficiency is the most common RBC enzyme deficiency which affects more than 400 million individuals worldwide (Cappellini & Fiorelli, 2008). More than 300 G6PD mutations have been reported to date, and most amongst them are the result of a single base pair change in the amino acid sequence of G6PD protein. G6PD deficiency is characterized in five classes based on clinical manifestations and residual enzyme activity, which are class I to V (WHO Working Group, 1989). Generally, G6PD deficient individuals remain asymptotic throughout their life except when exposed to certain drugs or with intake of certain food such as fava beans, resulting in hemolysis (WHO Working Group, 1989). The most common clinical symptoms of G6PD deficiency are hemolytic anemia, neonatal jaundice and chronic non-spherocytic hemolytic anemia (CNSHA) (Desnick et al., 2001).

The structure of G6PD exists in dimer or tetramer equilibrium based on electrostatic interactions, pH and ionic strength. The dimer has two subunits located along symmetrical β-sheets. Each monomer of G6PD has a catalytic binding site which contains substrate G6P and coenzyme NADP^+^ along with a structural NADP^+^ binding site located 15 Å away from catalytic site. This structural NADP^+^ site is present only in higher organisms (Kotaka et al., 2005) which play an important role in long term structural stability in human G6PD (Wang et al., 2008).

Point mutation at structural NADP^+^ site causes decreased enzyme stability leading to Class-I deficiency according to WHO classification exhibiting a clinical condition known as chronic non spherocytic hemolytic anemia (CNSHA). Mutations which cause severe G6PD deficiency other than CNSHA are categorized as class-II deficiency. The nature of the binding site of structural NADP^+^ indicates its structural importance in G6PD structure. Mutations other than class-I and class-II causing G6PD deficiency are away from the structural domain of G6PD protein. Studies show that point mutations in class I, class II and III have a low catalytic ability, decreased efficiency and less compact structure compared to wildtype G6PD (Gómez-Manzo et al., 2014; Au et al., 2000).

Mutations close to the binding site alter initial properties and interaction of G6PD with substrate and coenzyme NADP^+^, resulting in a change of thermodynamic properties and interaction of enzyme (Nguyen, Nguyen & Le, 2016).

More than 300 mutants of G6PD exist in nature; therefore, experimental studies for all mutants are deemed to be impractical. On the other hand, computational methods, in particular molecular dynamics simulation, are a robust and efficient tool for providing detailed insight into the dynamical properties of proteins and structural changes associated with them. The polymorphic nature of G6PD makes the enzyme a good candidate to explore the dynamical behavior and structural stability of its mutants with varying thermodynamics using molecular dynamics simulation. Only a few G6PD mutants have been previously studied via computational methods (Nguyen, Nguyen & Le, 2016; Doss et al., 2016); however, mutations in the dimer interface and their effect on the structural and functional properties of the G6PD enzyme still need attention to predict and design potent inhibitors against these mutants.

Here, we have focused on G6PD mutants with known pathological conditions which have mutations close to the dimer interface; to understand the effect of structural changes and their effects on substrate binding site associated with these mutations. Among the selected mutants A335T known as G6PD Chatham with 1003G>A cDNA substitution at Exon 9 lying at the βH- βI loop of the protein. This variant has been reported in Italy, Asia and Africa. According to the pathological condition it is categorized as class-II variant.

The second mutant R393H is known as Nashville, with 1178G->A cDNA substitution at exon 10 lying at dimer interface at β sheet βL. The variant is categorized in class-I based on severity of pathogenicity. This variant is reported in the USA, Italy and Portugal.

Mutant V394L, known as Alhambra, with 1180G->A cDNA substitution also lies at the dimer interface at β sheet βL adjacent to R393H. The mutation in the dimer interface causes severe clinical manifestation and categorized in class-I similar to R393H. This variant has been reported in Sweden and Finland (Minucci et al., 2012).

For these selected mutants, we have investigated the effect of amino acid mutations on 3D structures and structural flexibility of proteins is investigated using MD simulations of the wildtype and three mutants of G6PD. The objective of the work is to explore structural and dynamic effect of mutations that may act as guide for potential design of inhibitors to treat mutants for their normal functioning leading to cure of diseases associated with these mutations. Moreover the structural changes in enzymatic activities of the mutant are evaluated from the simulation results.

## Materials & Methods

### Model preparation

**Figure 1:**
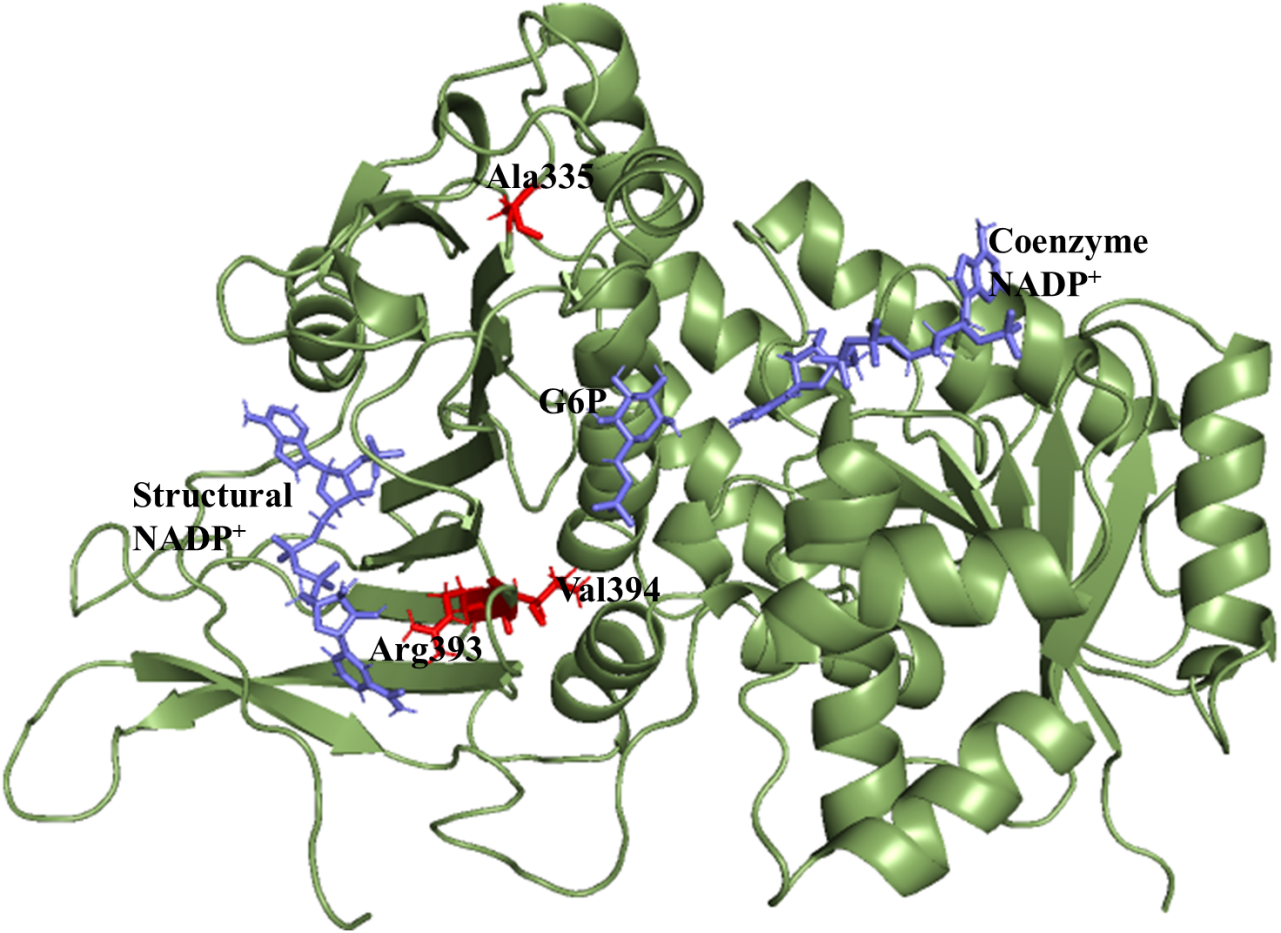
Modelled structure of G6PD enzyme. 3D model of G6PD. Ligand G6P, Coenzyme NADP^+^ and Structural NADP^+^ are represented in blue. Mutants are shown in red color.

X-ray crystallographic structures of wildtype G6PD enzyme complexed with G6P substrate, coenzyme NADP^+^, and structural NADP^+^ were retrieved from the protein data bank with PDB ID 2BHL and 2BH9 (Kotaka et al., 2005). The substrate, the coenzyme, and the structural NADP^+^ are present as ligands in the wildtype human G6PD for normal functioning. However; no crystal structure of the human G6PD enzyme is available so far, with the three ligands altogether in the form of a single structure in the protein data bank. To build the wildtype G6PD enzyme structure, we developed a model structure by superimposing both the 3D structures (PDB IDs: 2BHL and 2BH9) with the help of PyMol software (De Lano, 2002) (Fig. 1).

### Sequence alignment

UNIPROT and dBSNP databases were (accessed on Nov 2020) retrieved to obtain the sequence and position of amino acid mutations in wildtype G6PD. FASTA sequence of each mutant structure was retrived from dbSNP database. In order to identify the mutation position sequence alignment of above mentioned modeled structure of wildtype was carried out with mutant FASTA sequence (obtained from dbSNP database) using online T-coffee multiple sequence alignment tool (Notredame, Higgins & Heringa, 2000) via ClustalW (Sievers et al., 2011) with default parameter. To create mutant structure; In-silico site-directed mutagenesis was carried out at the corresponding mutation sites in the modeled wildtype G6PD structure using PyMol software (De Lano, 2002) choosing rotamer with minimum energy and clashes. Few of pathogenic mutations lying within binding site of structural NADP^+^ and dimer interface belonging to classes I and class- III) were chosen for this study Table 1.

**Table 1:** Selected variants dbSNP accession number, classes, amino acid position and change. Data represents dSNP accession number, classes, amino acid position and change in each amino acid. Column V, depicts each amino acid of enzyme replaced in mutants, R by H, V by L and A by T.

Ser	Accession no	Amino acid/mutation	Class	Mutation
1	rs137852316	393 (Nashville/Anaheim)	Class-I	R>H
2	rs137852335	394 (Alhambra)	Class-I	V>L
3	rs5030869	335 (Chatham)	Class-III	A>T

### Ligand modeling

Ligands were geometry optimized at Density functional (DFT) level of theory using B3LYP functional and 6-31G(d,p) basis set in Gaussian 09 (Frisch et al., 2016). From the optimized geometries ESP charge were calculated at HF/6-31G* level of theory followed by RESP charge in antechamber module of Amber 20 followed by parameterization of ligands using Generalized AMBER Force Field (GAFF) (Wang et al., 2004).

### Molecular dynamics simulation

All the protein structures were subjected to molecular dynamics simulation with the help of GPU based pmemd module implemented in the AMBER 20 simulation package. The protein structure was treated with the Amber 99SB-ILDN force field (Lindorff-Larsen et al., 2010), whereas the ligands (G6P, coenzyme NADP^+^, and structural NADP^+^) were parameterized with the generalized AMBER force field (GAFF). Initial configuration of the simulation systems was obtained with the help of the xLEaP module of the simulation package. The systems were protonated, neutralized with counter ions, and solvated with TIP3P water models thus resulting in a dodecahedron box under periodic boundary conditions.

#### Simulation protocol

The solvated systems were minimized in three steps to remove bad contacts. In the first step, the steepest-descent minimization was performed while applying a harmonic restraint of 25 kcal mol^−1^ Å^2^ on the solute for 100,000 steps following 10,000 steps of conjugate-gradient minimization. In the subsequent steps, restraints were gradually removed with a difference of 5 kcal mol^−1^ Å^2^ on the solute using a steepest-decent algorithm. In the final step, an unrestrained minimization of 100,000 steps was performed for the whole system. The minimizations were followed by a five-stage heating step for 40 ps each in which the temperature was raised from 0 to 300 K with a difference of 50 K while applying the harmonic restraint of 25 kcal mol^−1^ Å^2^ and removing 5 kcal mol^−1^ Å^2^ in each step with a time step of 0.5 fs in canonical (NVT) ensemble. This was then followed by 2 ns equilibration in isobaric-isothermal (NPT) ensemble at a constant pressure of 1 bar using Berendsen barostat (Berendsen et al., 1984) while keeping a harmonic contact of 25 kcal mol^−1^ Å^2^ on solute atoms that was subsequently removed with a difference of 25 kcal mol^−1^ Å^2^ (Ryckaert, Ciccotti & Berendsen, 1977). On getting well-equilibrated, production MD was performed for 100 ns for all systems in the NPT ensemble. During simulation, the Particle-mesh Ewald summation method was used to calculate electrostatic interaction (Darden, York & Pedersen, 1993) using the the cut-off of 1.4nm for both the electrostatic and van der Waals (vdW) interactions SHAKE algorithm was used to constrain bonds involving hydrogen atoms while setting the time-step of 2 fs for all the simulation. The sampling of the trajectories was carried out every 2 ps which were then processed for the analysis using CPPTRAJ package (Roe & Cheatham III, 2013) of AMBERTOOLS v.20. XMGRACE software program and R-studio were used to generate plots (Turner, 2005).

### Binding free energy calculation

The binding free energy was calculated using the Molecular Mechanics-Poisson Boltzman surface Area (MMPBSA) method (Homeyer & Gohlke, 2012; Miller III et al., 2012). The binding free energy of the complex is given as below (Miller III et al., 2012; Rastelli et al., 2010):

(1) \begin{eqnarray*}\Delta {G}_{bind}=\Delta {G}_{complex}- \left( {G}_{protein}+{G}_{ligand} \right) .\end{eqnarray*}

Total binding energy is the contribution of two thermodynamic quantities *i.e.*, ΔH and entropy ΔS (Salmas et al., 2015; Hou et al., 2011) as mentioned below:-

(2) \begin{eqnarray*}\Delta {G}_{bind}=\Delta H-T(\Delta S)\end{eqnarray*}

whereas enthalpy is the contribution of molecular mechanics energy and free energy of solvation as mentioned below (Rastelli et al., 2010)

(3) \begin{eqnarray*}\Delta H=\Delta {\mathrm{E}}_{MM}+\Delta {G}_{sol}\end{eqnarray*}

Δ*E*_MM_ signifies the molecular mechanics energy components of bonded and non-bonded forces of interactions (Verma et al., 2016)

(4) \begin{eqnarray*}\Delta {E}_{MM}=\Delta {E}_{\mathrm{bonds}}{+\Delta E}_{\mathrm{angles}}{+\Delta E}_{\mathrm{torsions}}+\Delta {E}_{vdW}+\Delta {E}_{\mathrm{electrostatics}}.\end{eqnarray*}

Molecular mechanics energy is the contribution of energy of bonded terms *e.g.*, bonds, angles, dihedrals and non-bonded energy van der Waals and electrostatic energy. For protein ligand complex the contribution of free energy due to bonded terms are excluded by taking it zero so Eq. (4) is modified as:-

(5) \begin{eqnarray*}\Delta {E}_{MM}=\Delta {\mathrm{E}}_{vdw}+\Delta {\mathrm{E}}_{elec}\end{eqnarray*}

The ΔE_*elec*_ contributes to the polar contribution of solvation free energy and non-polar energy (Rastelli et al., 2010; Salmas et al., 2015; Hou et al., 2011)

(6) \begin{eqnarray*}\Delta {G}_{sol}=\Delta {G}_{polar}+\Delta {G}_{non-polar}.\end{eqnarray*}

Contribution of polar energy can be calculated using Poison Boltzmann model whereas nonpolar contribution can be calculated using the following equation:

(7) \begin{eqnarray*}\Delta {\mathrm{G}}_{non-polar}=\gamma SASA+\mathrm{\beta }\end{eqnarray*}

where *γ* and β (Homeyer & Gohlke, 2012; Salmas et al., 2015; Hou et al., 2011; Verma et al., 2016) are effective surface tension parameter and offset value respectively.

### MM-PBSA calculations

To find out binding free energy, the single MD trajectory approach (Homeyer & Gohlke, 2012; Srinivasan et al., 1998) was applied using holo trajectory to extract snapshots of complex, ligand and protein. Five hundred frames were extracted throughout the simulation. PBSA program in amber was used employing Parse atomic radii (Sitkoff, Sharp & Honig, 1994; Syeda Rehana & Zaheer, 2018). Salt concentration of 0.150 and GB model with igb = 2 with mbondi2 was used. The surface tension (*γ*) value was fixed to 0.005 kcal/ (mol Å^2^). The solvent probe radius of 1.4 Å was used to calculate the SASA.

## Results

### Molecular dynamics simulations

**Figure 2:**
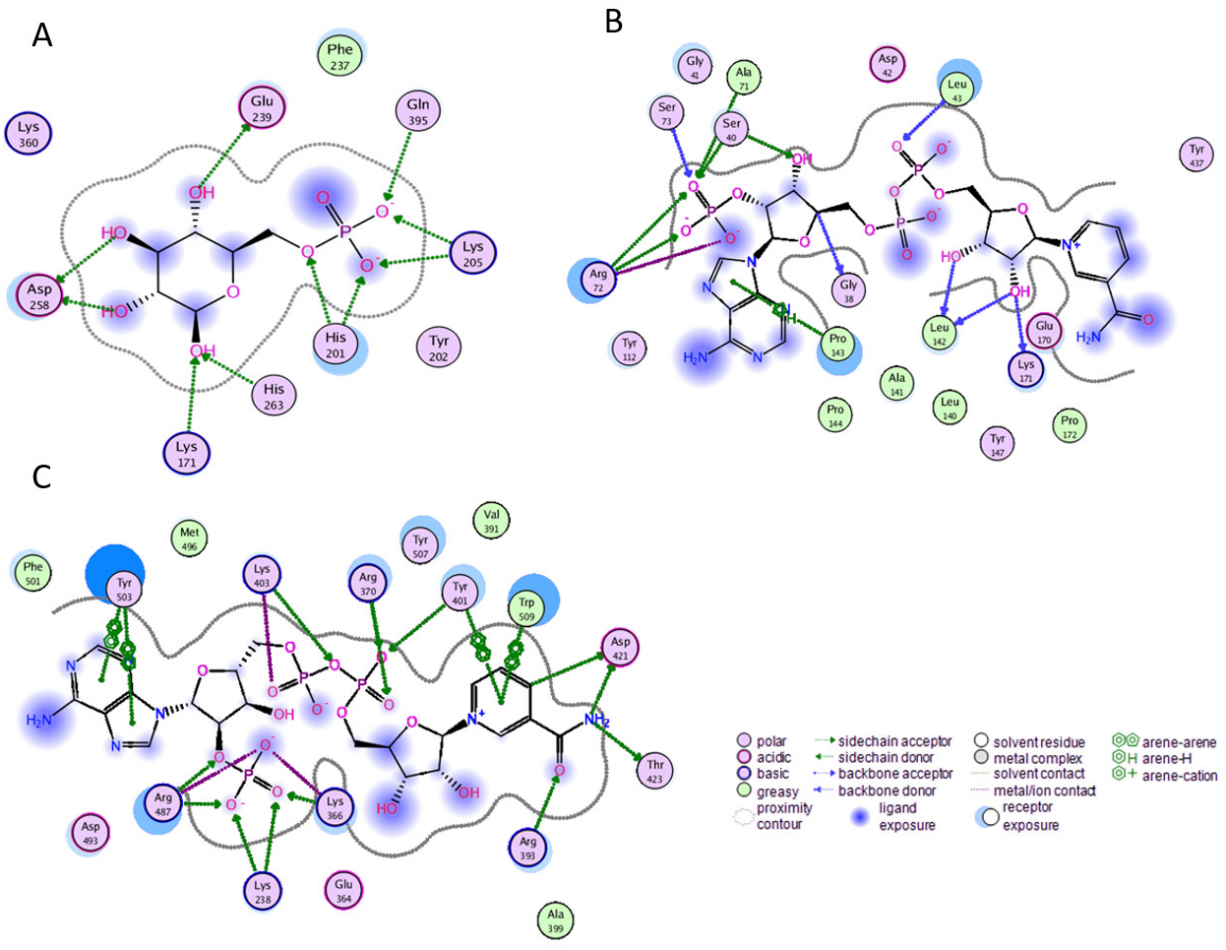
Ligand interactions of G6PD enzyme. 2D ligplot showing interactions of G6PD in binding sites with (A) G6P, (B) coenzyme NADP+, and (C) structural NADP+ interacting with residues of G6PD.

Before performing molecular dynamics simulation we calculated protein-ligand interaction profiles to know the key residues involved in the binding of ligand with protein and to get an insight whether these interactions would be maintained after MD simulation as a result of the effect of mutation on these interaction. Ligand interaction profile of the ligands, G6P and NADP^+^ with the neighboring residues of wildtype G6PD enzyme is depicted in Fig. 2. G6PD enzyme provides three binding sites such as the site in the catalytic domain for the substrate (G6P) binding, the site for the coenzyme NADP^+^ binding in close proximity to the G6P site and the structural NADP^+^ binding, which is away from the catalytic domain. The binding site for the structural NADP^+^ provides enhanced stability to the enzyme structure (Wang et al., 2008). Figure 2A illustrated G6P binding site in which Gln395, Lys205 interacted with the phosphate group of G6P via donor–acceptor interactions, whereas Lys171 belonging to a conserved peptide and His263 interacted with the hydroxyl group of G6P via hydrogen bonding. Figure 2B displayed interaction plot of the coenzyme NADP^+^ with G6PD residues in which Arg72 interacted with the 2′-phosphate via hydrogen bonding, and Pro143 formed arene-arene interaction with the adenine ring of NADP. The main chain residues *i.e.*, Gly41 and Asp42 form hydrogen bond with adenine ribose 3′-hydroxyl and the bisphosphate O atoms of the coenzyme NADP^+^ (Fig. 2B). Structural NADP^+^ binding site is represented in the Fig. 2C. As seen in the Fig. 2C, at the structural NADP^+^ binding site; Arg393 interacted with the nicotinamide group of the structural ligand, and Tyr401 and Tyr503 formed *π*–*π* stacking interaction with the nicotinamide and the adenine ring, respectively. Lys238, Lys366 and Arg487 interacted with the 2′ phosphate group through donor–acceptor interactions. Arg393, Glu239 and Pro396 are also found to be involved in the binding and defining shape of the binding site. The interaction profiles of these three ligands provided a static picture of the enzyme-ligand interaction to depict how the ligands attained stability in the binding site; however the dynamics of these interactions needed to be evaluated in detail through the simulation studies.

#### Effect of mutations on residual interactions

**Figure 3:**
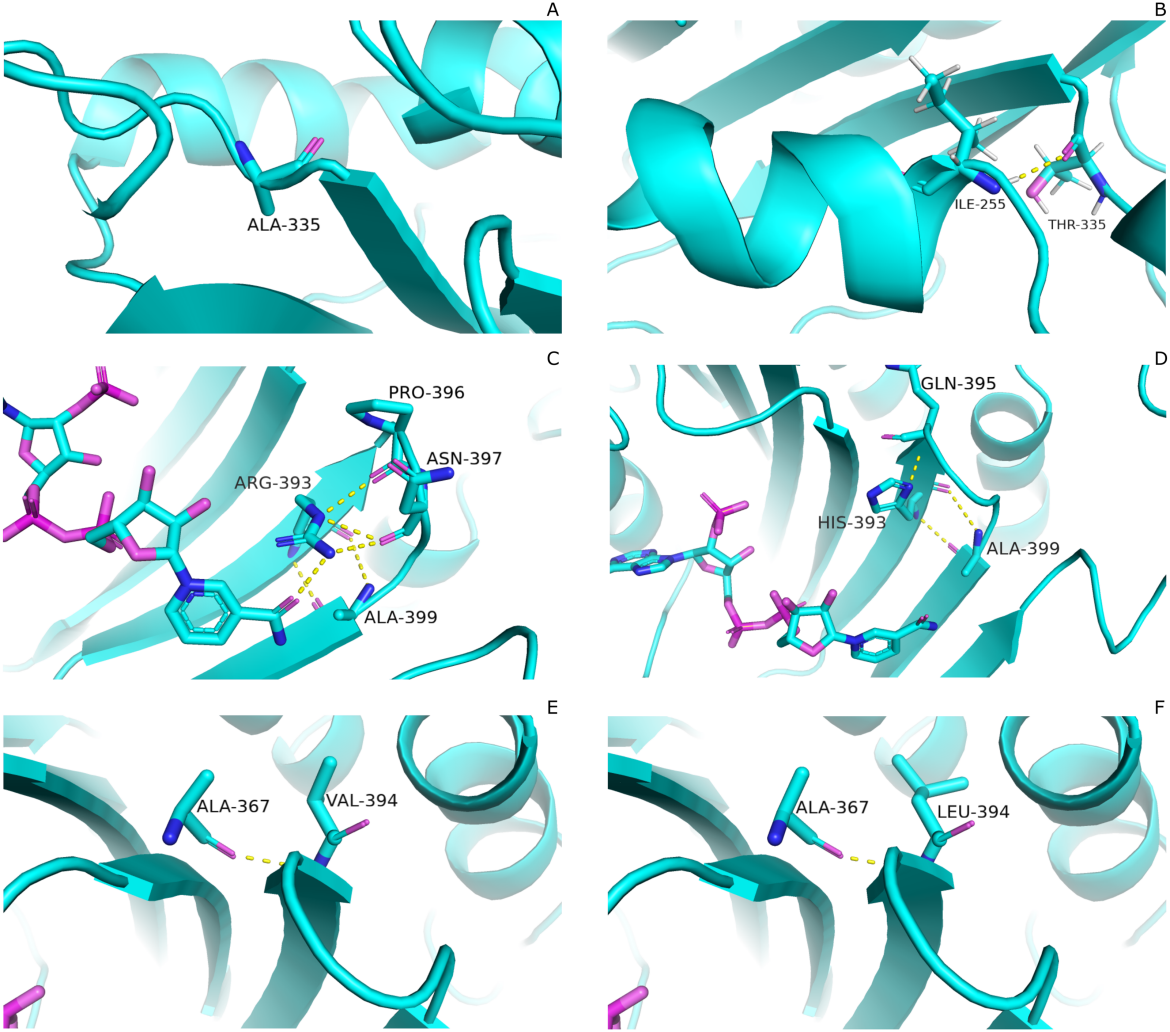
Comparartive analysis of interaction G6PD and mutant amino acids. (A) Wild type 335, (B) Mutant 335T, (C) Wild type 393R, (D) Mutant 393H, (E) Wild type 394V, (F) Mutant 394L. Mutants show change in the interaction pattern of neighboring residues.

Mutations have pronounced effect on residual interactions of enzyme. Comparison of residual contact pattern for wildtypes and mutants have been depicted in Fig. 3. A335T lies at the end of βi of an extensive nine stranded β-sheet which forms the part of dimer interface accommodating structural NADP^+^. Alanine in wildtype is the part of loop region at the end of extensive β-sheet network close to dimer interface (Fig. 3A). Introduction of large sized threonine instead of alanine resulted in the formation of polar contacts with Ile 255 (Fig. 3B) in adjacent αi- helix.

Based on the severity of pathogenicity; the mutants in class-I of G6PD are characterized into two categories *i.e.*, lying in structural NADP^+^ binding site and at dimer interface. R393H and V394L fall in both of these categories. Figure 3C depicted that guanidino group of Arg393 in wildtype interacted with amide oxygen of nicotinamide ring of structural NADP^+^, Asn397 and Ala399, whereas in mutant R393H; replacement of Arg393 with Histidine resulted in the loss of these interactions. Histidine developed polar interaction with Gln395 and Ala399 (Fig. 3D). Loss of charge interaction may have resulted in the movement of nicotinamide ring of NADP^+^ away leading to loss of key residue interactions.

Similarly; V394L lies in the binding site of structural NADP^+^ being the part of large extended β-sheet region. In wildtype backbone contact of Valine 394 with Ala 367 is clearly observable according to Fig. 3E, however this contact of Ala 267 was maintained with Leucine as well in mutant V394L (Fig. 3F). Part of the β-sheet and αf helix are enriched with the hydrophobic side chain interactions between Ile392, V394, and V400 with M207, L211 and L214. In V394L; Incorporation of larger Leucine in V394L as compared to smaller sized Valine may have displaced the adjoining residues causing week hydrophobic interactions and hence higher flexibility of this region. As a consequence, perturbation in αf helix may occur which is involved in the substrate binding and hence protein stability. Valine is a Cβ branched hydrophobic residue which may not be involved in catalysis however; its neighboring residue R393 is involved in forming hydrogen bond interaction with nicotinamide ring of structural NADP^+^. There may be a change in the interactions at structural NADP^+^ binding site with subsequent movement of NADP^+^ resulting in overall change in dynamics.

#### Structural stability of wildtype G6PD and its mutants

**Figure 4:**
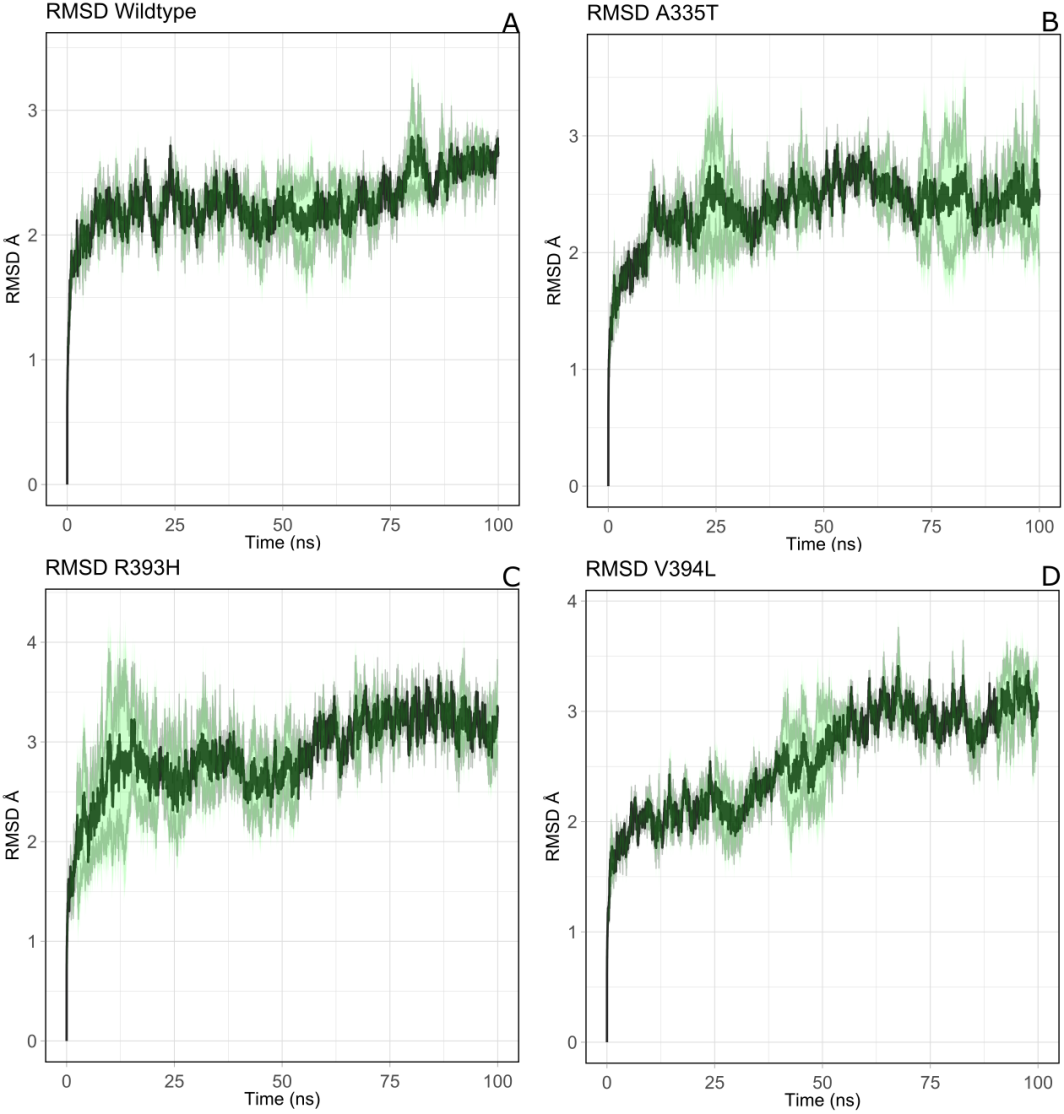
The comparative backbone RMSD of wild type and mutants in duplicate simulation. Run1 is shown in black and run2 shown in red color, (A) wildtype, (B) A365T, (C) R393H, (D) V394L. The reference structures of RMSD calculation were the starting structure of MD simulations.

Overall structural stability of wildtype complex was accessed by calculating root mean square deviation (RMSD) of C α atoms during the course of 100 ns simulation. Duplicate runs were performed and the average RMSD was found to be 2.5 Å as shown in Figs. 4A–4D. The system was equilibrated between 30 ns to 50 ns for first run. There was an increase in RMSD between 50 ns to 80 ns up to 3.2 Å after which it was equilibrated from 80 ns to 100 ns. In duplicate simulation the system was equilibrated with an average RMSD of 2.11 Å. Overall average RMSD of the two structures was 2.5Å (Fig. 4A). No significant difference in stability of the two structures was observed which clearly indicated the reliability of results and enough stability of structure. The stable structures were further selected for comparison of stability between wildtype and its three mutants *i.e*; A335T, R393H and V394L via RMSD of their backbone atoms. For the clarity of differences between the structure of wildtype and mutants, residual contacts are presented in Fig. 3.

For the mutant A335T both runs seem to get equilibrated with an average RMSD of 2.6 Å Both systems tend to get stabilized between 80 ns to 100 ns with an RMSD of 2.1 Å. Overall average RMSD of the two structures was 2.3 Å (Fig. 4B). In mutant R393H; a similar trend was observed in which the system shows stability between 40 ns to 60 ns with an average RMSD of 2.52 Å for both of the runs. A gradual increase in RMSD was observed at 80 ns for both structures which tend to stabilize with RMSD value of 2.8 Å at 100 ns. Overall average RMSD of the duplicate structures was 2.4 Å as depicted in the Fig. 4C. In V394L RMSD was found to be 3.2 Å on average. Both runs exhibited stability between 60 ns to 80 ns after which a fluctuation was seen for both of the structures with an average RMSD value of 3.1 Å (Fig. 4D).

The variation in RMSD signifies the conformational changes in the protein structure happening throughout the simulation. As it is evident from the Fig. 4A, backbone RMSD of wildtype G6PD fluctuated in the range between 2.0 to 2.5 Å, however; it was converged at an average value of 2.5 Å. On the other hand, the RMSD of A335T ranging from 2–2.8 Å converged at an average of 2.5 Å depicted in the Fig. 4B, however, R393H ranging from 2.5–3.3 Å converged at an average of 2.8 Å (Fig. 4C). RMSD of V394L ranged between 2.0 to 3.5 Å and converged at an average of 3.0 Å as evident from the Fig. 4D. Overall there was a slight difference in RMSD of wild type and mutant A335T. Also, RMSD plot of R393H AND V394L showed variation in RMSD as compared to wildtype. A slight difference in global RMSD of A335T may be attributed to the position of mutation away from the binding site of substrate and coenzyme indicating that the mutation did not alter significant change in protein stability. RMSD of R393H and V394L was higher as compared to wildtype providing an indication that mutation of essential residues in the binding site of structural NADP^+^ could be ascribed to the low stability of the enzyme structure. In R393H; Arginine with electrically charged side chain has been replaced by Histidine both serving as basic residues. The replacement of guanidine group of Arginine with imidazole ring of Histidine resulted in the change of interactions with neighboring residues as shown in Figs. 3C–3D however; the effect would not be so pronounced to show a prominent change in the global RMSD.

In the same way, in V394L Valine having small side chain has been replaced with Leucine. Both residues belong to the same class of amino acids where the side chain is hydrophobic. The larger size of Leucine as compared to Valine may have changed the local distances of binding site resulting in a change in overall RMSD of the protein as shown in Fig. 4D.

The validation of changes in structural stability was further evaluated by the computation of averaged root mean square fluctuation (RMSF).

#### Residual conformational changes and associated flexibility

**Figure 5:**
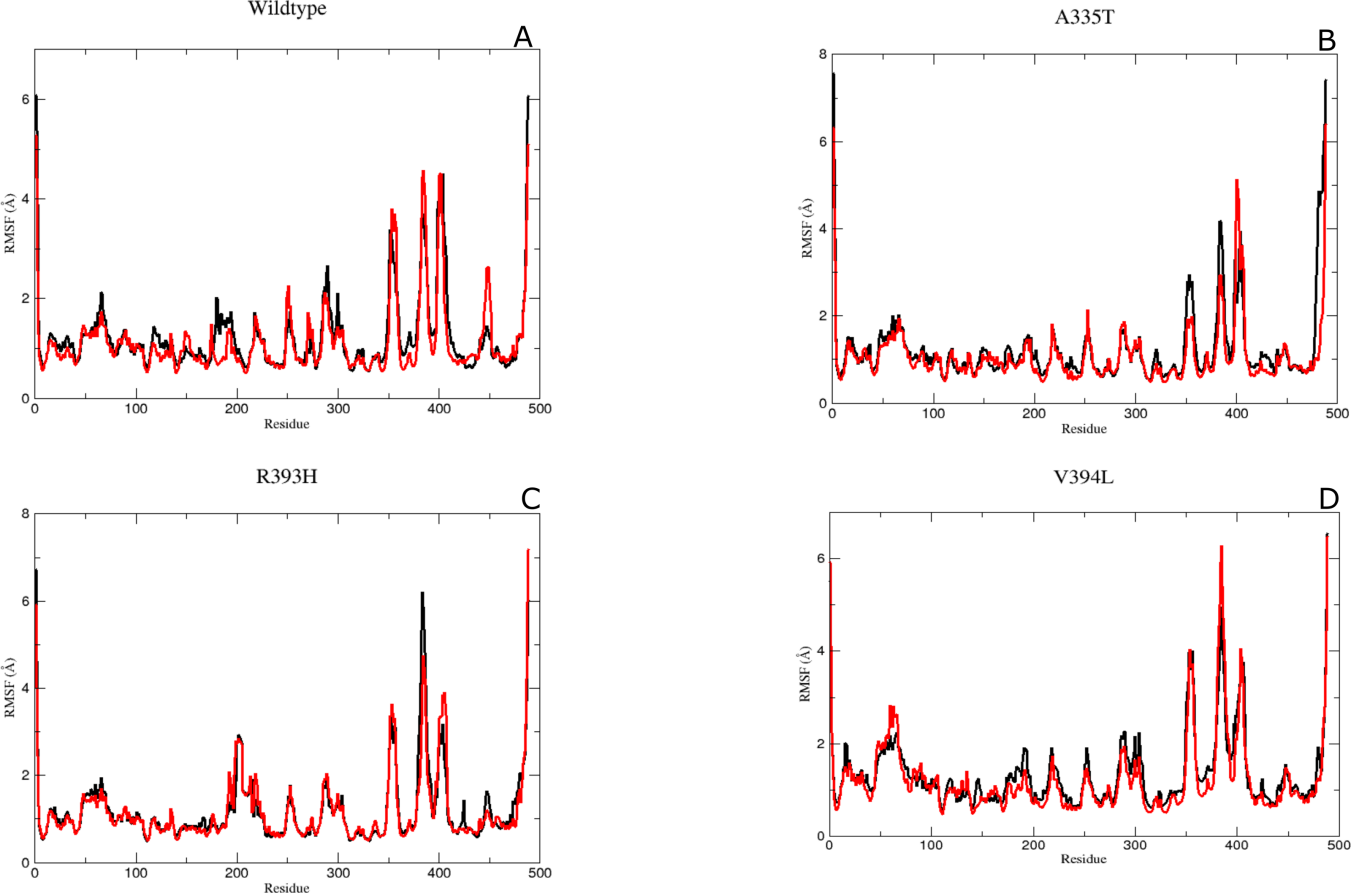
The comparative Backbone RMSF of wild type and mutants in duplicate simulation (run1 shown in black and run2 shown in red color) for flexibility analysis. (A) Wildtype, (B) A365T, (C) R393H, (D) V394L. The comparison of both the runs show the same trend in RMSF depicting reliability of simulation.

It was assumed after the preliminary RMSD calculations that mutations of the essential residues may lead to changes in the residual dynamics of the enzyme which were assessed as averaged RMSF for the backbone atoms of wild type and its mutants. Proteins in loop region are more flexible than rest of secondary structure elements. In order to evaluate the flexibility of G6PD enzyme; RMSF of the two duplicate simulations were compared and displayed in Figs. 5A–5D. RMSF plot disclose that loop regions in G6PD exhibited greater flexibility and randomness as compared to the other secondary structural elements.

Residue region 298–305, 350–358 and 384–386 representing loop region exhibited higher RMSF values for both of simulations in wildtype (Fig. 5A). Similarly the loop region corresponding to residues 190–197 exhibited higher flexibility. This region is adjacent to conserved residue peptide (198–205) which is involved in substrate binding. Residue region 426–431 representing loop region and connecting the large βM-sheet with αI helix and residues 488–489 representing loop region of-c-terminus tail show higher fluctuation with RMSF value of 5.5 Å. Residue region 350–358 signified the loop region connecting two βL and βK.380-405 region exhibiting βM sheet showed RMSF values of 2.8 Å and 5.8 Å respectively revealing flexible nature of residues in this region of protein. This region belongs to the β + α domain of G6PD accommodating structural NADP^+^ binding site. Higher RMSF may indicate the mobile nature of the region.

RMSF peaks for mutant A335T were comparable to the wildtype indicating the perseverance of flexibility of G6PD enzyme. *i.e*., RMSF of the region harboring residues 350–358 exhibited value of 2.5 Å and 4 Å for residues 385–401 revealing the flexibility of these regions was not affected by mutation (Fig. 5B).

The RMSF plot for R393H and V394L (Figs. 5C & 5D) showed higher flexibility for the loop region connecting the βM-sheet with αI helix *i.e.*, 6Å as compared to wildtype. Interestingly the residue region involving the substrate binding *i.e.*, 198–201 exhibited higher flexibility in V394L as compared to wildtype.

Overall an increase in flexibility showed the instability of substrate binding site residues.

#### Flexibility of Residues in the binding site

Mutants R393H and V394L showed a decrease in flexibility for dinucleotide binding finger print region containing residues 38–44 demonstrating that these mutants did not affect the binding of coenzyme NADP^+^ site.

The residue region belonging to the conserved peptide *i.e.*, 198–206 plays an important role in the substrate binding *e.g.,* His 201 is involved in stabilizing the G6P ring in proper orientation for catalysis. RMSF value for this region in wildtype and A335T was 1.8–1.9 Å as compared to the RMSF of R393H and V394L which was 2.5 Å. This difference manifested that mutation resulted in the increase in the flexibility of binding site residues in mutant R393H and V394L (Figs. 5A–5D)

##### Substrate interaction with protein and variation in residual contacts.

**Figure 6:**
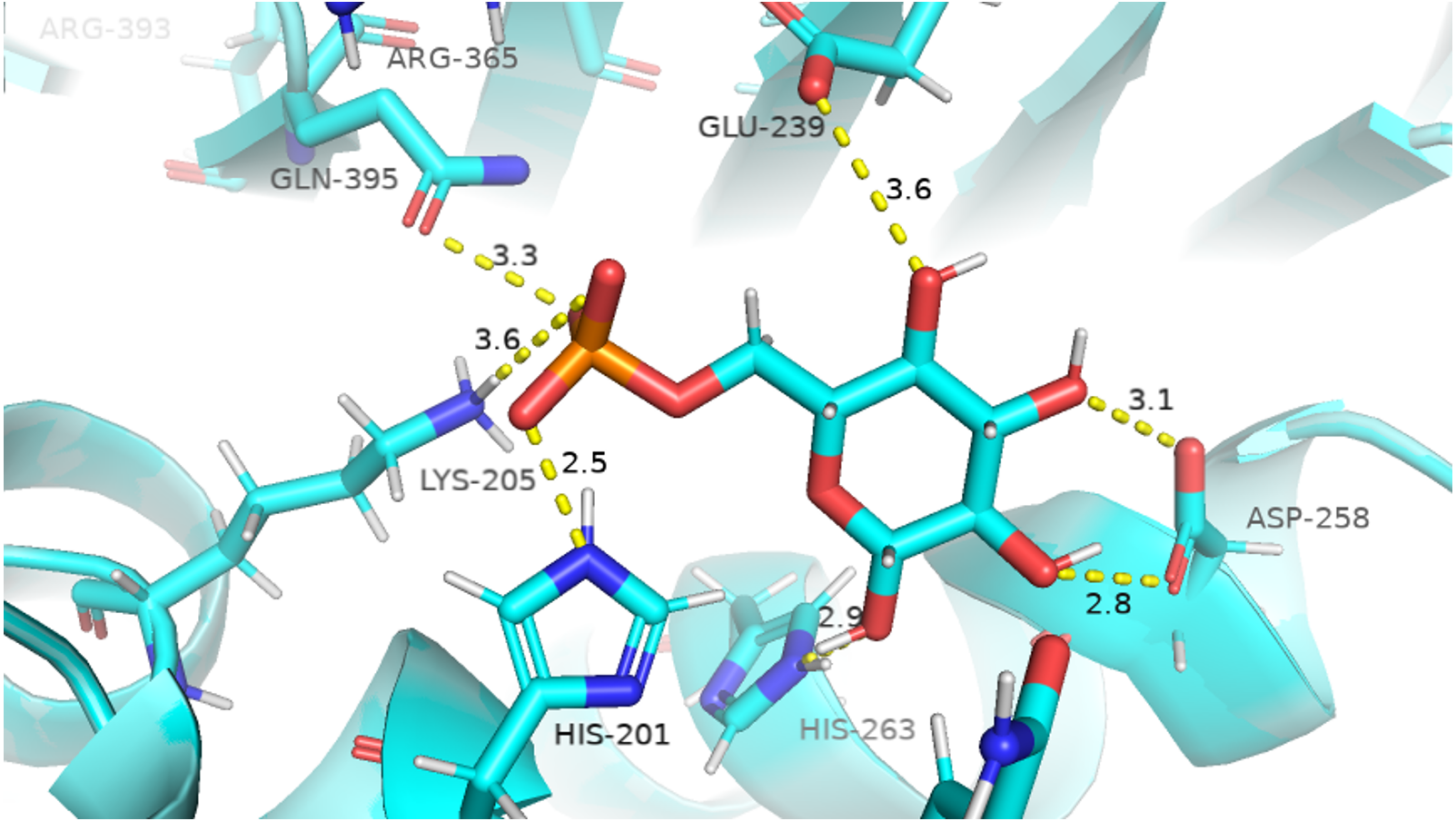
Native contacts between G6P and residues in the binding site. Native contacts between G6P and residues in the binding site. The native contacts were calculated based from crystal structure.

**Figure 7:**
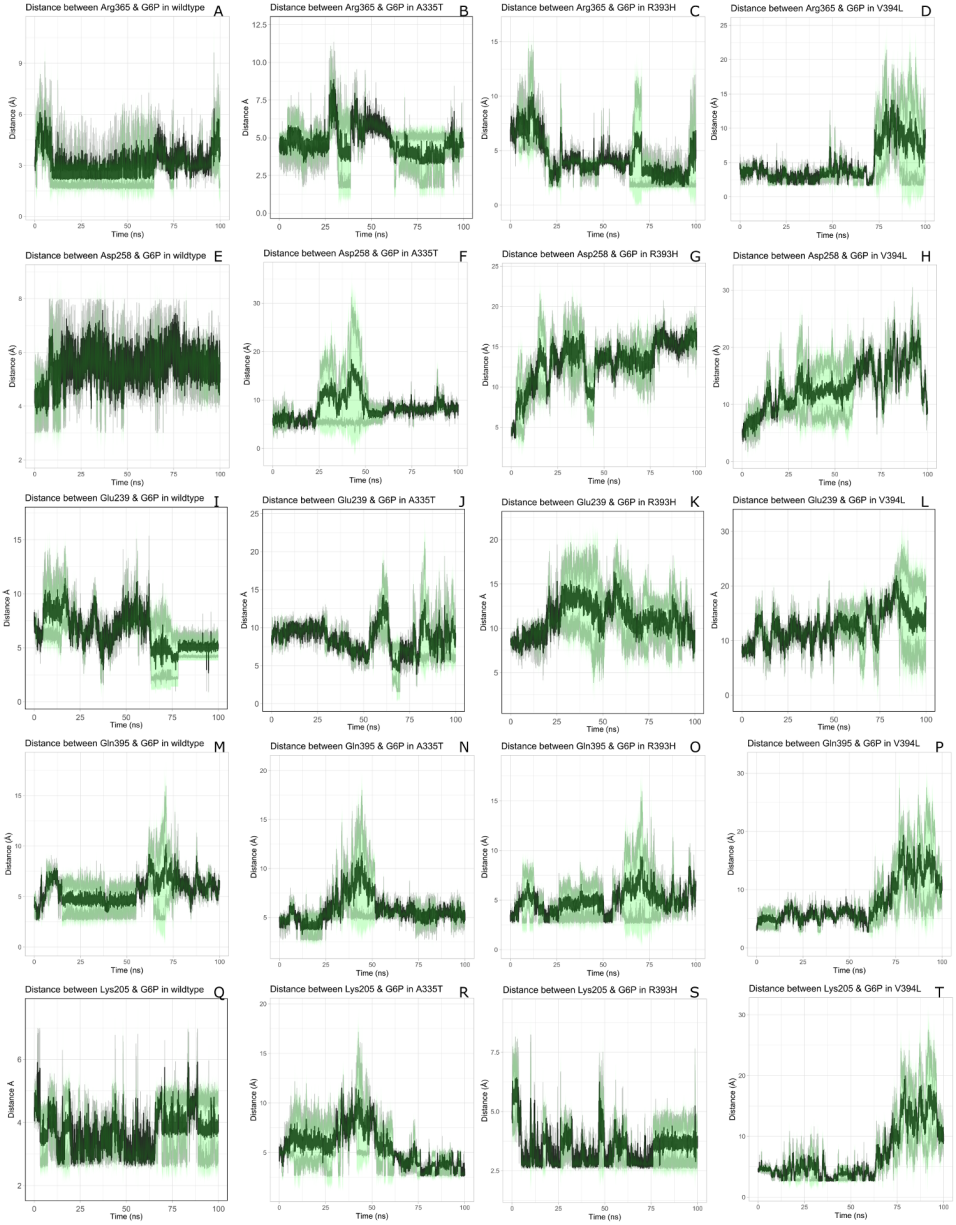
Time evolution of the distance of G6P substrate. Time evolution of the distance of G6P substrate with the key interacting residues in the binding site of wild type and mutant. The shaded lines represent individual run while dark green line show the average of the two runs.

The visual description of interactions of substrate G6P with the neighboring protein residues in crystal structure are depicted in Fig. 6. To describe the changes in dynamics of these interacting residues during the simulation; we compared the time evolution of average distance of residues in binding site of G6P in wildtype and mutants. (Fig. 7).

The interactions of G6P with neighboring residues were preserved in wildtype during the course of simulation. As it is apparent from the Fig. 6 that Arg 365 interacts with O3 atom of G6P in crystal structure. The time evolution graph of interaction of G6P with Arg365 for wildtype and mutants shown in Figs. 7A–7D. It is apparent from the Fig. 7A that in wildtype, at the start of simulation the average distance was 4 Å till 10 ns which gradually reduced to 3 Å until 60 ns. Upon reaching 100 ns the average distance maintained itself to 4 Å. In A335T this distance gradually increased from 5 Å to 7.5 Å till 50 ns after which it reduced to 6 Å on average as evident from the Fig. 7B. Likewise the average distance in distance R393H was found to be 4.5 Å between 20 ns to 60 ns and 70 ns to 90 ns however; fluctuations in the average distance up to 7.5 Å was observed from 0 ns to 20 ns. Also these fluctuation went up to 6 Å between 60 ns–65 ns and 80 ns–100 ns showing destabilization of hydrogen bond distance (Fig. 7C). In V394L the average distance remained up to 4.5 Å till 70 ns and increased up to 10 Å till 100 ns (Fig. 7D).

Average distances of Asp 258 and G6P is manifested in (Figs. 7E– 7H) for wildtype and mutants respectively. Asp 258 in the binding site of G6P interacts with O5 and O4 atom of the G6P ring in crystal structure as depicted in Fig. 7E. In the wildtype the average distance remained 5 Å during 100 ns of simulation. A fluctuation in distance was observed for all of the three mutants *i.e.*, in A335T the average distance of 7 Å was maintained, In R393H and V394L the distance increased up to 12 Å during the simulation according to Figs. 7F–7H respectively. The fluctuations in the distances between residues in the binding sites with substrate in mutant structures showed that mutations resulted in perturbation of native residual contacts resulting in the overall less stable structure.

Figures 7I–7L demonstrated average distances of Glu 239 with G6P. As Fig. 6 indicated that Glu 239 interacts with O7 atom of the G6P in the crystal structure, a deeper analysis of Fig. validated that this contact was preserved in the wildtype during course of simulation with an average distance of 3.5 Å  (Fig. 7I). In mutant A335T; a large fluctuation in distance was observed during simulation between 20 ns to 40 ns with an average of 15 Å which was reduced to an average of 4.5 Å from 50 ns to 100 ns indicating that the hydrogen bond was not preserved during some parts of simulation. Moreover A335T showed some conformational change in the larger loop movement as well (Fig. 7J). For R393H, Fig. 7K revealed that large fluctuation in distance were found with an average value of 7.5 Å. For mutant V394L initially this distance was 10 Å which gradually increased up to 15 Å from 75 ns to 100 ns indicating the movement of β-sheet away from the substrate (Fig. 7L).

Average distances between Gln395 and G6P were also calculated for wildtype and mutants and represented in Figs. 7M– 7P). In the crystal structure of wildtype, the Gln395 interacts with O atom of G6P. This interaction was sustained till 55 ns after which a change in distance was observed between 60 ns to 70 ns which gradually reduced to 5 Å for wild type (Fig. 7M). This distance was increased up to 6.5 Å between 0 ns to 50 ns for mutant A335T t after which it was stabilized (Fig. 7N).

For mutant R393H, Fig. 7O signifies an increase in average distance to 7.5 Å  manifesting that change in structural NADP^+^ site resulted in the displacement of key G6P binding site residues as well. For V394L the distance between G6P and Gln 395 was 5 Å till 60 ns after which a gradual increase was observed with an average distance of 15 Å from 60 ns to 100 ns (Fig. 7P) Residue Lys205 from the conserved peptide region interact with the O2 atom of the phosphate group of G6P. Average distance of Lys205 with G6P are demonstrated in the Figs. 7Q–7T) for wildtype and mutants. Though this contact has been preserved in the wildtype with an average distance of 3.5 Å as reflected through Fig. 7Q. However; in A335T the distance increases from 5 Å to 7 Å till 50 ns after which a gradual decrease was observed maintaining a distance of 4 Å  (Fig. 7R). Correspondingly, a fluctuation in the distance was observed in R393H from 0 ns to 70 ns after which it was maintained up to 3.5 Å  (Fig. 7S). The distance was not preserved for the mutant V394L as the simulation proceeded with a gradual increase to 10 Å between 75 ns–100 ns according to the Fig. 7T.

**Figure 8:**
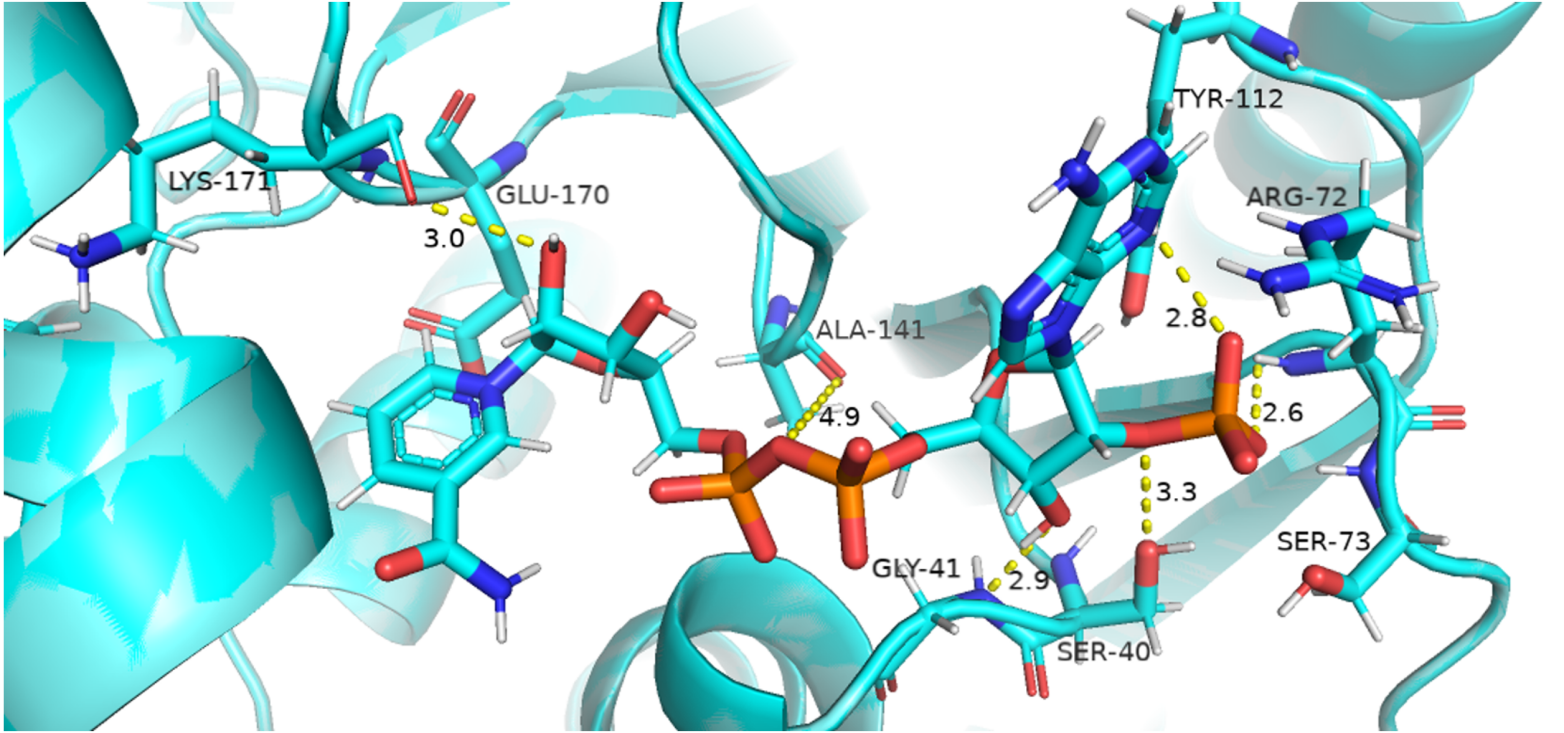
Native contacts between Coenzyme NADP^+^ and residues in the binding site. The native contacts of *Coenzyme NADP*^+^ were calculated based from crystal structure.

##### Coenzyme NADP^+^ interaction with protein and variation in residual contacts.

**Figure 9:**
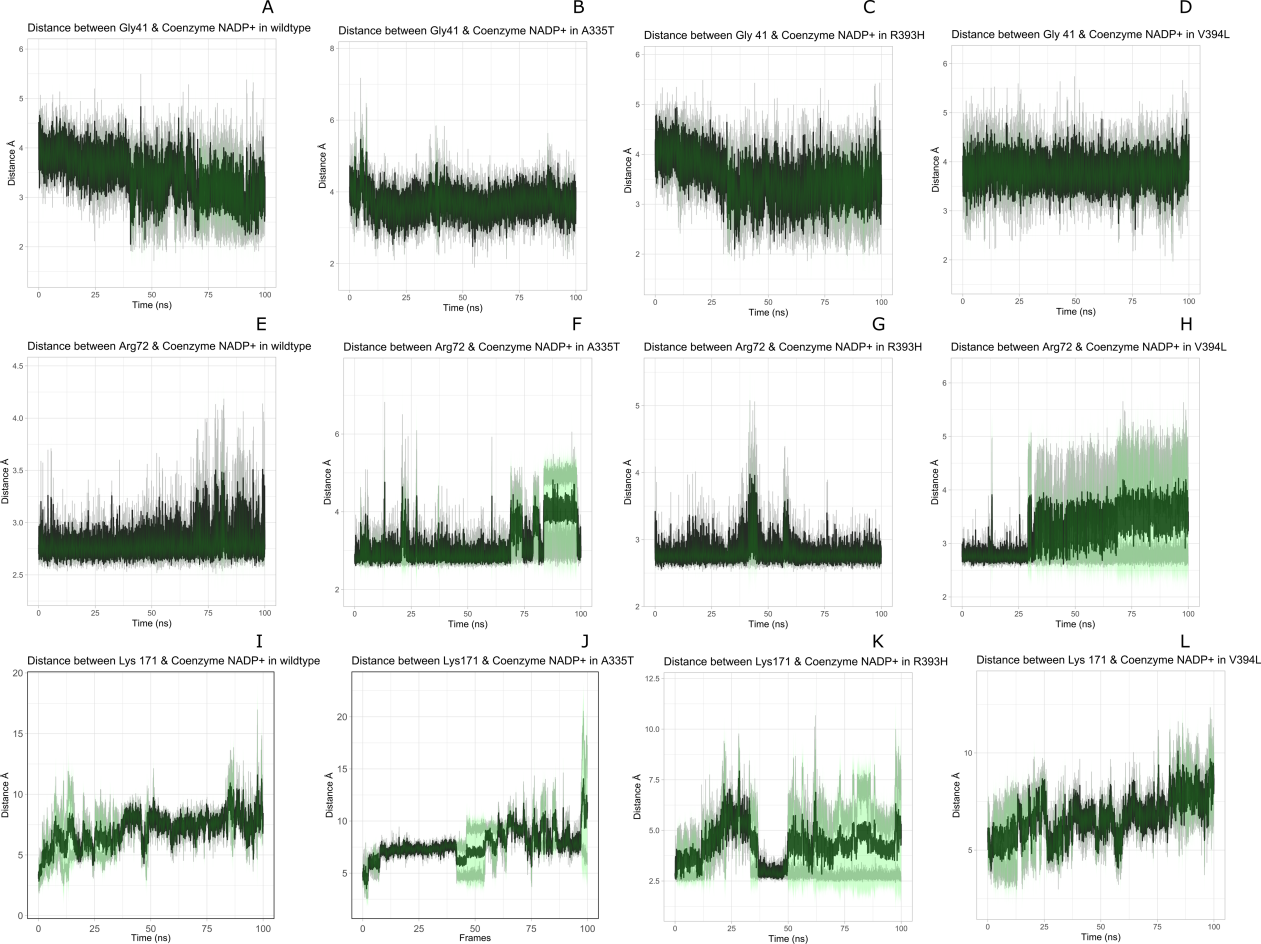
Time evolution of distance of Coenzyme NADP^+^ with the key interacting residues in the binding site for wild type and mutant (A–L). Insignificant change in the distances indicate that mutations did not have significant alterations co-enzyme NADP^+^ site.

Residual contacts between key interacting residues and coenzymes NADP+ are described in the Fig. 8 and time evolution of key residues interaction with the cofactor NADP^+^ were calculated as depicted in the Fig. 9.

Figure 8 presented that in crystal structure Gly 41 of the nucleotide binding finger print region (GASGDLA) form hydrogen bond with 3′ hydroxyl group of NADP^+^. Average distances of interacting residues with coenzyme NADP^+^ are depicted in Fig. 9.

Average distances of reside Gly41 with NADP+ for wild type, mutants A335, R393H and V394L are depicted in the Figs. 9A– 9D) respectively. For wildtype the distance was reduced from 4 Å to 3.5 Å gradually during the course of simulation. Importantly; this distance of coenzyme NADP^+^ remained conserved with an average value of 3.8 Å in A335T. For mutant R393H the average distance was 4 Å initially at 0 ns which gradually reduced to 3.5 Å. An average distance of 3.5 Å was observed in V394L.

Interactions of Arg72 with NADP^+^ for wildtype and mutants are evident from Figs. 9E–9H. Guanidino group of Arg72 and 2′phosphate of NADP^+^ form hydrogen bond. This interaction was maintained for wildtype with at an average distance of 2.5 Å. In A335T the distance was 3 Å till 70 ns after which a peak was observed from 70 ns to 95 ns with an average value of 4 Å. In R393H, this distance remained 2.9 Å with a peak between 40 ns to 45 ns. In V394L the average distance was 2.9 Å which increased gradually from 25 ns to 100 ns with an average value of 3.5 Å.

2′ hydroxyl group of nicotinamide ribose form hydrogen bond with the Lys 171 of the βE- αe turn in crystal structure (Fig. 8). The average distance for the wildtype was calculated as 7 Å. In A335T average distance of 6.5 Å was maintained till 50 ns which gradually increased up to 10 Å with large fluctuations. In R393H a fluctuation in the distance was observed between 0 ns to 30 ns from 3 Å to 6.5 Å which gradually reduced to 3 Å. In V394L a gradual increase in distance ranging from 5 Åto 10 Å was observed during the course of simulation indicating the loop movement away from the cofactor. Lys 171 is the second residue of conserved peptide which keeps the integrity of binding site by interacting with ring O atom of G6P. A change in distance in mutant V394L indicate that conformational change at this region may occurred in the binding site resulting in the increase in distance.

##### Structural *NADP*^+^ interaction with protein and variation in residual contacts.

**Figure 10:**
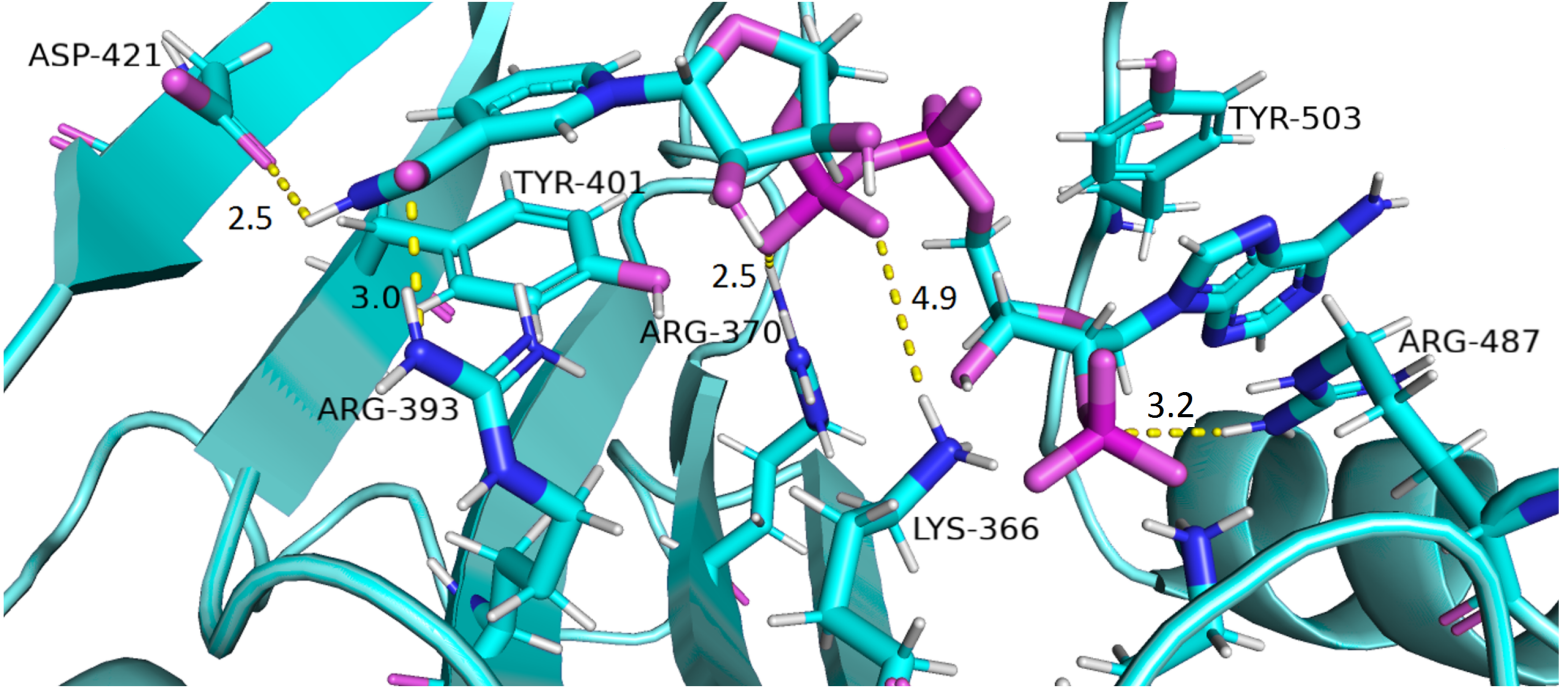
Residue interactions within binding site of Structural NADP^+^. The native contacts for interactions within binding site of Structural NADP^+^ were calculated based from crystal structure.

**Figure 11:**
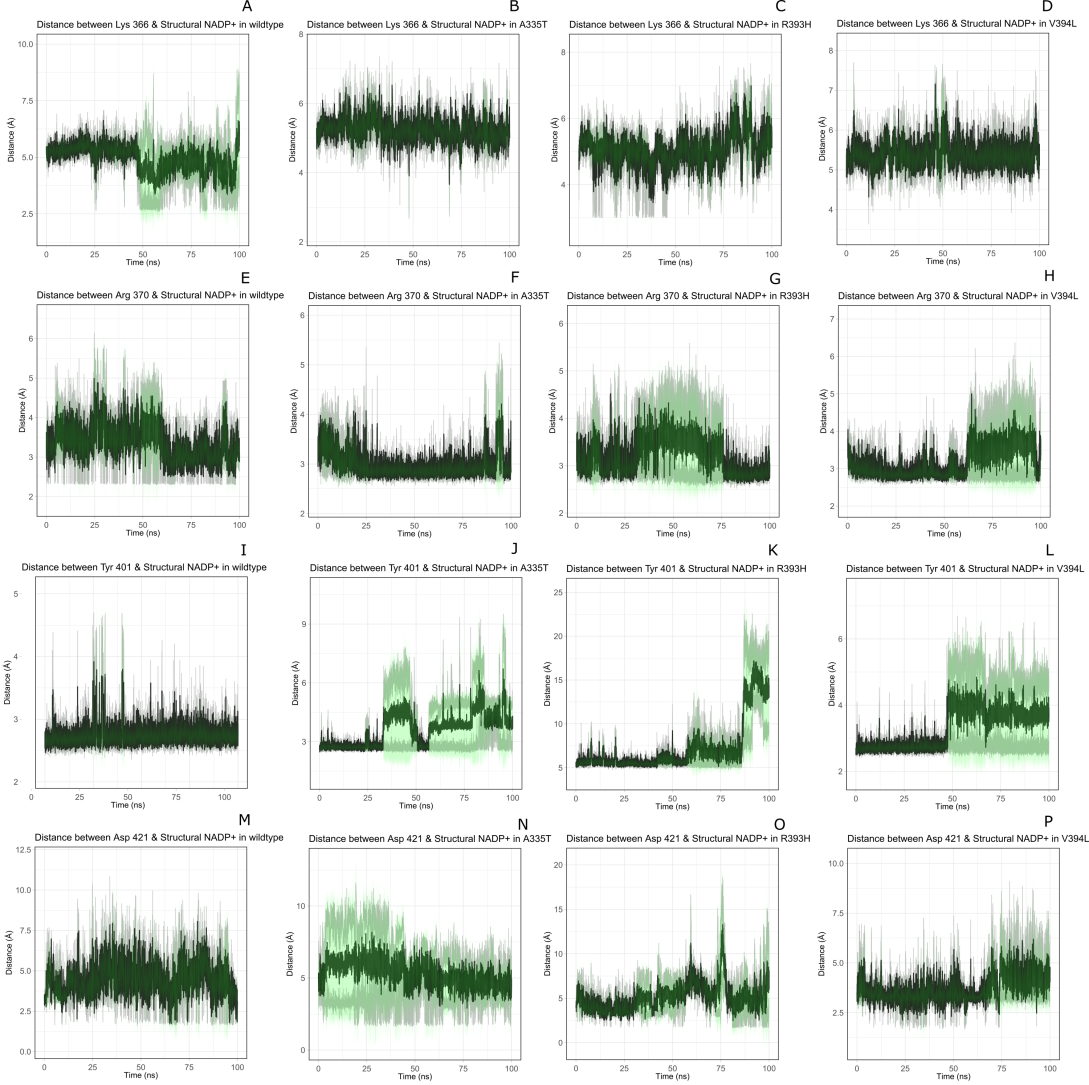
Time evolution of distance of structural NADP^+^ with the key interacting residues in the binding site for wild type and mutant (A–L). Changes in the distances depict the change in interaction of mutants with protein in the structural NADP^+^ binding site.

Interaction of protein residues Tyr 401, Asp 421, Lys 366 and Arg370 with structural NADP^+^has been represented in Fig. 10 and their distances indicated in the Fig. 11.

Lys 366 interconnects with 2′ phosphate group of structural NADP^+^ in crystal structure. The average distance of 5.5 Å was observed in wildtype from 0 ns to 50 ns which gradually reduced to 5 Å. Figure 9A. On average distance of 5.5 Å was observed for both mutants *i.e.*, A335T and R393H however R393H showed higher fluctuation from 75 ns to 100 ns. A similar trend in distance was observed for the mutant V394L where the average distance was 6 Å from 0 ns to 25 ns and gradually reduced to 5.5 Å. Displacement of structural NADP^+^ due to loss of hydrogen bond with Asp 421 and Try 401 oriented 2′ phosphate closer to the Lys 366 in R393H with a decrease in average distance of 3.5 Å.

Arg370 interact with the bisphosphate of via hydrogen bond in crystal structure. In wildtype the average distance of 3.0 Åwas maintained as compared to mutant A335T 3.3 Å. In R393H the average distance was 6 Å which increased from 30 ns to 80 ns to 7.5 Å. In V394L the distance remained 3.5 Å till 60 ns which increased up to 3.7 Å till 100 ns. R393H showed loss of hydrogen bond as a result of which increase in distance was observed.

Tyr 401 form *π*–*π* stacking interaction with nicotinamide ring of NADP^+^ in crystal structure. This interaction was conserved in wildtype and mutant A335T, V394L with an average distance of 2.5 Å between Oxygen of bisphosphate and hydroxyl group of Tyr401. However this *π*-stacking interaction was lost with the adenine ring of NADP ^+^ in R393H due to the increase in the distance.

Asp 421 forms the polar interaction with amide hydrogen of nicotinamide in crystal structure. Average distance for the wildtype was 4.5 Å. In A335T the average distance was higher *i.e.*, 6 Å till 40 ns which gradually reduced to 5 Å till 100 ns. In R393H the average distance increases from 5 Å to 7 Å during the course of simulation due to movement of adenine ring of NADP^+^ away resulting in loss of this bond. In V394L the distance was 4.5 Å till 70 ns after which it increased up to 5 Å. The average bond length of this interaction was 8 Å in mutant R393H. Asp 421 lies at the center of dimer interface. Loss of interaction in the R393H results in the displacement of nicotinamide ring of NADP^+^ away from the histidine.

In wildtype guanidine group of Arginine 393 form hydrogen bond with 2′ hydroxyl ribose ring of structural NADP^+^ as depicted in Fig. 3C. Mutation from Arginine to Histidine resulted in the loss of this hydrogen bond Fig. 3D.

#### Effect of mutations on conformation and catalysis

**Figure 12:**
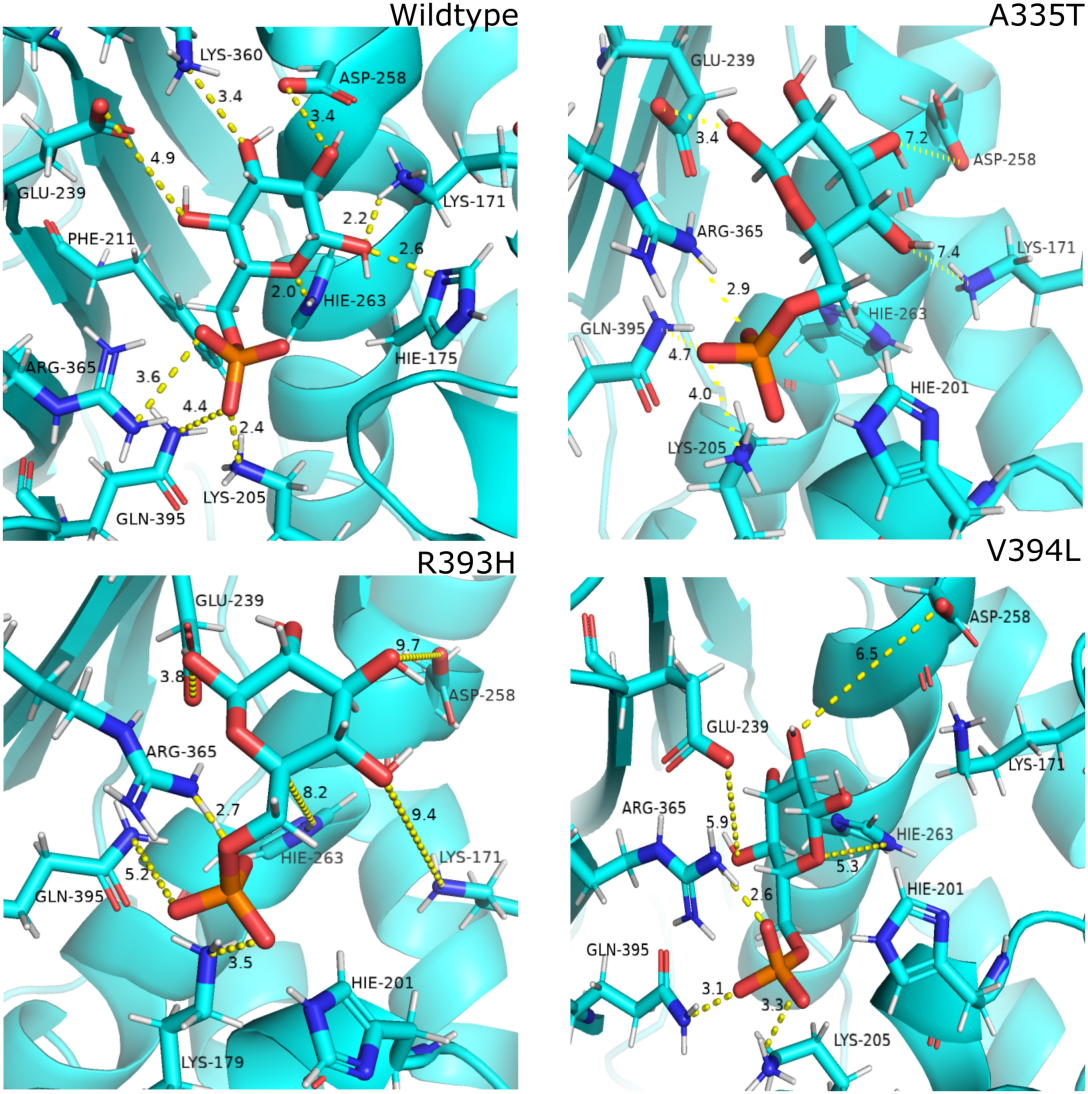
Most common centroid structure showing mean change in distances in G6P binding site. (A) Wildtype (B) A335T, (C) R393H, (D) V394L. Variation in interactions of G6P with neighboring residues were observed in mutants as compared to wildtype.

As described earlier that A335T lies at the end of βi of extensive 9 stranded β-sheet which forms the part of dimer interface accommodating structural NADP^+^. Alanine in wildtype is the part of loop region at the end of extensive β-sheet network close to dimer interface. Introduction of large sized threonine instead of alanine resulted in the formation of polar contacts with Ile 255 (Fig. 3B) in adjacent αi- helix. Resultantly; loss of α helix structure in to loop at residue region containing amino acids 252–254 EFG took place making this region more flexible. The adjacent region of αi- helix showed disordered helix containing residues 258–263 which play an important role in substrate binding *i.e.*, Asp 258 is involved in the formation of polar contacts with ring oxygen atoms of G6P. Due to increase in flexibility the distance between G6P and Asp 258 increases resulting in the movement of Asp 258 away from the binding site. Similarly; Orientation of G6P has also been changed resulting in the movement of ring O atom away from the His 263 considered to be involved in the catalysis.(Fig. 12B)

Guanidino group of Arg393 in wildtype interacted with amide oxygen of nicotinamide ring in structural NADP^+^, Asn397 and Ala399 Fig. 3C. In mutant R393H; replacement of Arg393 with Histidine resulted in the loss of these interactions (Fig. 3D). Loss of charge interaction resulted in the movement of nicotinamide ring of NADP^+^ away leading to loss of *π*- *π* stacking interaction of Tyr 401 and Tyr 509 with nicotinamide ring of NADP^+^ as depicted in Fig. 12C. Similarly interaction between nicotinamide ring of NADP^+^ with Asp 421 and Arg 370 with oxygen atom of bisphosphate (Figs. 8B–8C) was lost resulting in the movement of nicotinamide ring away from the key interacting residues between substrate and NADP^+^ making the substrate binding site more flexible due to which substrate may have lesser stability in the binding site.

Based on the severity of pathogenicity the mutants of G6PD are characterized into two categories *i.e.*, lying in structural NADP^+^ binding site and at dimer interface. R393H and V394L fall in both of categories. V394L lies in the binding site of structural NADP^+^ being the part of large extended β-sheet region. Incorporation of larger Leucine in V394L as compared to smaller sized Valine in wildtype have changed the local contacts. Valine is a C β branched hydrophobic residue which is not involved in catalysis however it is directly attached to the neighboring residue R393. R393 is involved in forming hydrogen bond interaction with nicotinamide ring of structural NADP^+^. A change in the structure have resulted in the change of interactions in structural NADP ^+^ binding site with subsequent movement of residues in the binding site resulting in overall change in dynamics.

#### Most populated cluster conformation

In order to measure the interactions of substrate G6P, Coenzyme NADP^+^ and structural NADP^+^; a map of interactions of most populated cluster was produced after cluster analysis for wildtype as well as mutants. There was a variation in the distance of the binding site residues with substrate and cofactors for wildtype and mutants. Figure 12 describes the interaction established in the most populated cluster in the presence of G6P in wildtype and mutants (Fig. 12A). G6P develops hydrogen bond with Lys360, Asp258, Lys171, Arg365, Lys205 and Hie 263. A variation in the distance between these residues was observed upon mutation, *i.e*. loss of hydrogen bond with Asp 258, Lys 171 was observed for A335T as evident from the Fig. 12B. In R393H; loss of hydrogen bond took place for Asp25, Lys 171, Gln395, Hie 263 (Fig. 12C) and in V394L hydrogen bond was lost between G6P and Asp258, Glu 238, Gie263, Arg365, Lys171 as apparent in the Fig. 12D.

**Figure 13:**
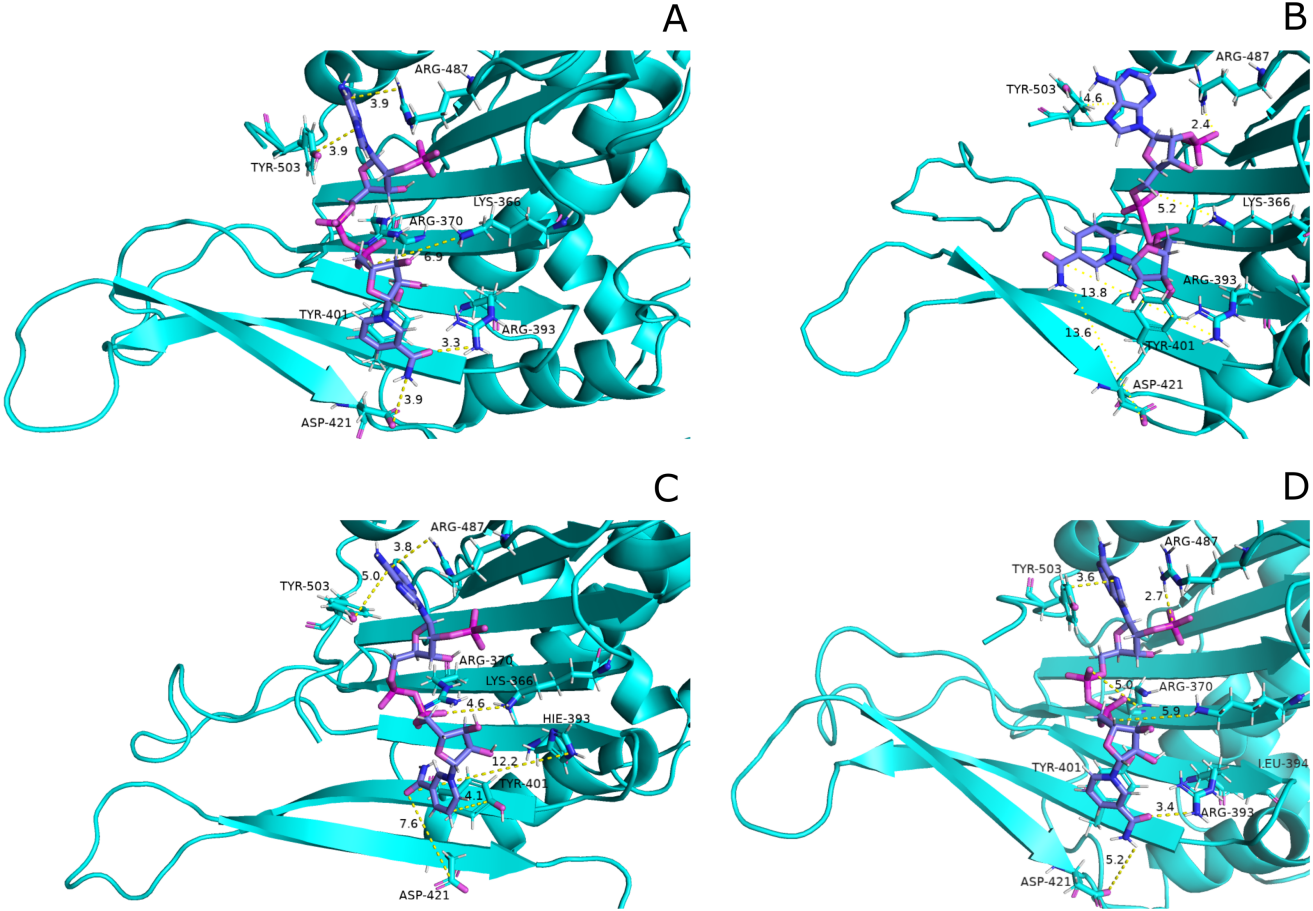
Most common centroid structure showing mean change in distances in structural NADP^+^ binding site. (A) Wildtype (B) A335T, (C) R393H, (D) V394L. Significant changes in interactions of nicotinamide ring with neighboring residues were observed in mutants as compared to wildtype.

These variations were also observed in the form of change in interactions in structural NADP^+^ binding site specifically in the nicotinamide ring region indicate the formation of hydrogen bond between nicotinamide ring of structural NADP^+^ with Arg393 and Asp421(Fig. 13A). Similarly; *π*-stacking interaction was developed between Tyr 401 and nicotinamide ring. Π-staking interaction was also maintained between Tyr503 and adenine ring of structural NADP ^+^. The *π*-stacking interactions were lost in mutants A335T, R393H and V394L in addition to the loss of hydrogen bond with Arg393 and Asp421 (Figs. 13B–13D) indicative of the fact that the change in binding mode of key residues in structural NADP^+^ binding site had influenced G6P binding site as well upon mutation.

### Secondary structure analysis

**Figure 14:**
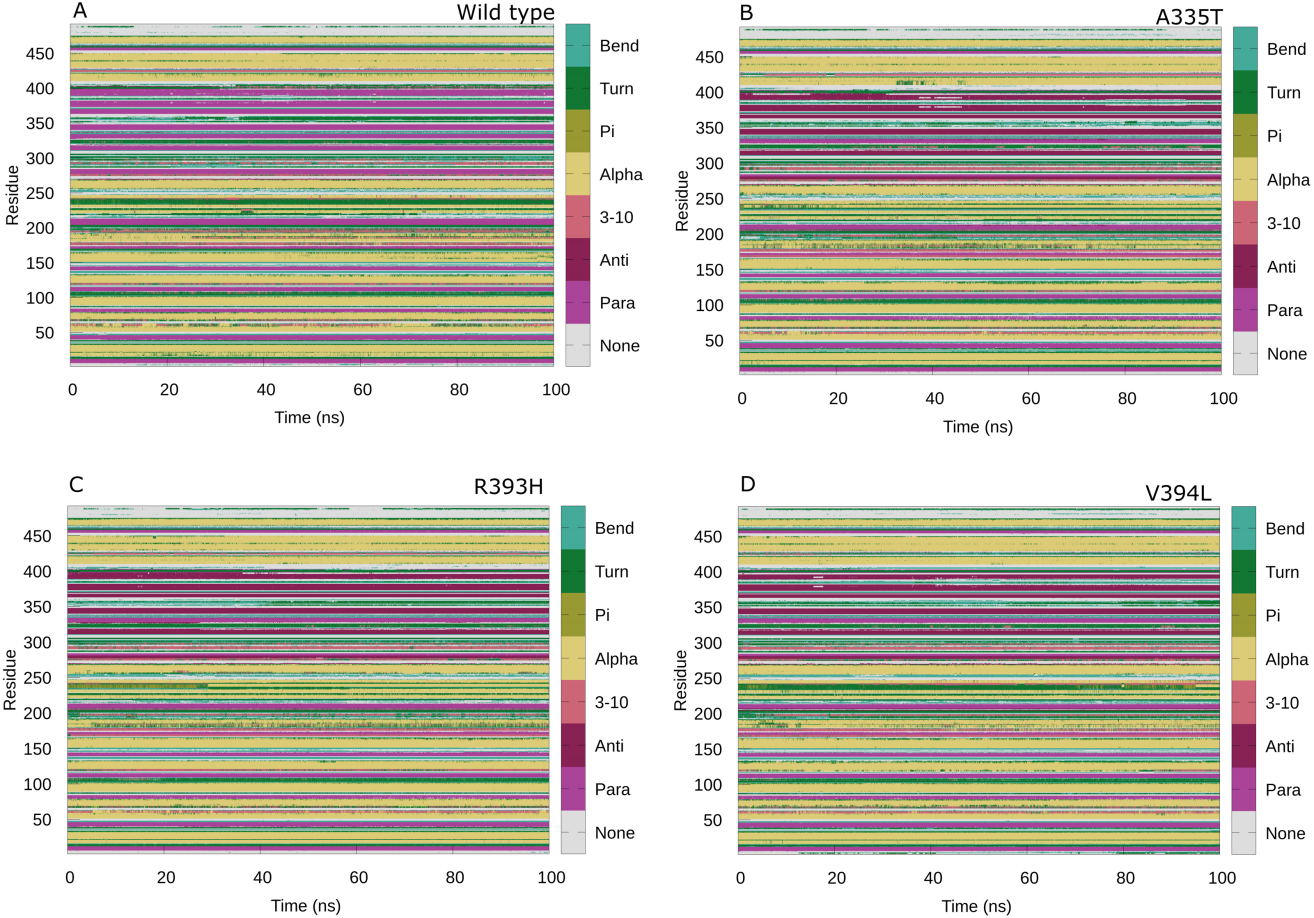
Protein secondary structure analysis. Secondary structure analysis of wild type G6PD and its mutant as a function of time from 0 to 100 ns. (A) Wildtype, (B) A335T, (C) R393H, (D) V394L.

Maintenance of secondary structure is crucial for studying the dynamic behavior of protein. Secondary structure analysis of the G6PD enzyme and its mutants was performed to monitor the structural changes associated with mutation as compared to wildtype in structural elements such as alpha helices, beta sheets, and coils during the simulation period. Trajectories at 50 ns and 100 ns were collected and compared to the structure at the first frame to investigate the structural changes during the simulation. Overall structural changes throughout the trajectory are represented in the Fig. 14. Small secondary structural changes associated with amino acid and their position is depicted in Table 2. In A335T change of secondary structure elements from alpha helix to turn for residues 410–415 of β + α domain was observed between 30 ns to 50 ns during the course of trajectory (Fig. 14B). In mutant R393H loss of loop took place for residues 230–240 starting from 40 ns to 100 ns and 30 ns to 100 ns as evident from Fig. 14C. Alike change in the structure was observed at 90 ns in V394L according to Fig. 14D. These residues belong to the β + α domain of G6PD; the structural NADP^+^ lies in the β + α domain. The perturbation in this region may have an effect on NADP^+^ binding.

**Table 2:** Changes in secondary structure and associated amino acids at 50 and 100 ns. Secondary structural analysis represents amino acid position, their sequence and structural change as a result of mutations at 50 ns and 100 ns.

**Variants**	**Time (ns)**	**Position**	**Amino acid Sequence**	**Structural change**
Wild type	50	ii. 300-301	ANN	Loop to turn
	100	i. 193-195	EDQ	Loop to turn
		ii. 201-203	RDN	Loop to turn
		iii. 300-301	ANN	Loop to turn
A335T	50	i.219-221	LMVLRF	α-helix to loop
		ii. 219-221	RIF	Turn to loop
	100	i. 144-146	TVY	Loop to turn
		ii. 316-319	EAT	Loop to turn
		iii.321-323	YLD	Loop to turn
		iv. 252-253	EF	*α*-helix to loop
R393H	50	i.85-87	EPF	*α*-helix to loop
		ii.144-146	PTV	Turn to loop
		iii. 219-221	RIF	loop to turn
		iv. 427-429	RYKN	Loop to turn
		v. 379-381	DIF	Loop to turn
	100	i. 300-302	ANN	Loop to turn
		ii. 379-381	DIF	Loop to turn
		iii. 448-451	SQMH	Loop to turn
		iv. 316-318	EAT	Loop to turn
V394L	50	i.221-225	FGPI	*α*-helix to loop
	100	i.221-225	FGPI	Loop to turn
		ii.156-158	ESC	Loop to turn

The change in secondary structure elements in this region represented that mutation had effected dynamical behavior of structural NADP^+^binding site thus affecting the structural stability of mutants. No significant difference in secondary structure was observed as evident from previous experimental studies (Gómez-Manzo et al., 2014) however; few local changes in secondary structures were observed throughout the simulation indicating the loss of stability as shown in Table 2 and Figs. 14A-14D.

### Binding free energy calculation by MM-PBSA

Binding free energy calculations for interaction of substrate G6P with wildtype and its mutants was done by MM-PBSA. The decomposition of binding free energy into its components has been shown in the Table 3 depicting the electrostatic energy component being the major contributor of binding fee energy. The Molecular mechanics energy (E_MM_) constitutes electrostatic and van der Waals energy components. The calculated E_MM_ for wildtype was −188.5 kcal/mol and; −147.5 kcal/mol, 138.8 kcal/mol, −150.7 kcal/mol for A335T, R393H and -V394L respectively. The molecular mechanics energy of wildtype indicated its structure stabilization as compared to mutants.

**Table 3:** Binding free energy values calculated by using MMPBSA method. Binding free energy components included electrostatic and vdW energy in kcal/mol. The total solvation free energy which is the blend of polar and non-polar energy terms.

Free Energy Component	Amber output term	Wildtype	A335T	R393H	V394L
		Energy (kcal/mol)	Energy (kcal/mol)	Energy (kcal/mol)	Energy (kcal/mol)
		Mean ± Std.dev	Mean ± Std.dev	Mean ± Std.dev	Mean ± Std.dev
ΔE_electrostatic_	EEL	−210.2695 ± 36.0636	−200.8102 ± 45.7379	−116.6845 ± 48.1122	−86.5013 ± 47.0398
Δ*E*_vdw_	VDWAALS	−8.2111 ± 4.9705	−3.2591 ± 4.2250	−4.1440 ± 3.9698	−7.5739 ± 3.5425
Δ*E*_MM_ = Δ*E*_electrostatic_ + Δ*E*_vdw_		−218.4806	−204.0693	−120.8285	−94.0752
**MM-PBSA**					
Δ*G*_polar_	EPB	180.2649 ± 27.5577	180.3975 ± 40.7232	100.5207 ± 41.2799	74.9024 ± 43.4364
Δ*G*_non−polar_	ENPOLAR	−14.4928 ± 2.8196	−11.0225 ± 2.2161	−10.4887 ± 2.8433	−15.786 ± 1.6303
Δ*G*_sol_ = ΔG_polar_+ Δ*G*_non−polar_		165.7721	169.375	90.032	59.1164
Δ*G*_polar_ + ΔE_electrostatic_		−30.0046	−20.4127	−16.1638	−11.5989
ΔGnonpolar solvation= ΔEvdw+ ΔGnon-polar		−22.7039	−14.2816	−14.6327	−23.3599
ΔH= ΔE_MM_ + ΔG_sol_		−52.7085	−34.6943	−30.7965	−34.9588
Total Binding Energy MM(PBSA)		−25.8260 ± 13.3498	−16.4031 ± 8.1141	−12.7896 ± 8.5373	−8.4411 ± 7.3496

The total solvation free energy which is the blend of polar and non-polar energy terms was unfavorable for all structure *i.e.*, wildtype, A335T, R393H and V394L.

The total electrostatic contribution (ΔG_polar_ + ΔE_electrostatics_) was least for the wildtype *i.e.*, −25.6 kcal/mol and highest for V394L having value −9.2 kcal/mol displaying lesser affinity of V394L for the substrate binding as compared to wildtype and other mutants. The impact of vander Waals energy together with the non-polar solvation free energy (ΔE_vdW_ + ΔG_non-polar_) constituted the non-polar energy term, which considerably contributed to the overall binding free energy by a value of −28 kcal/mol for the wildtype and for mutants being A335T; −27.1 kcal/mol, R393H; −16.3 kcal/mol and V394L; −28.8 kcal/mol. The net binding enthalpy (ΔH = ΔE _MM_ + ΔG_sol_) for wildtype was found to be −83.5 kcal/mol, as compared to A335T; −47.5 kcal/mol, R393H; −23.3 kcal/mol and V394L; −38 kcal/mol in which the molecular mechanics van der Waals (ΔE_vdW_) energetic contribution is the most dominating enthalpic factor driving protein-ligand binding. It is evident from the results of binding free energy calculations that substrate complexes with mutants have structural stability much lower than that of wildtype protein (Table 3). Also mutations in the wildtype have induced a change in substrate binding pattern leading to structural distortion of mutants.

## Discussion

The structural stability of protein is directly correlated to its function. Mutation can induce a stabilizing or destabilizing effect resulting in the change of physicochemical properties of mutants. G6PD deficient red blood cells face the challenge of low stability of already synthesized G6PD as well as unavailability of a nucleus to synthesize new enzymes to replace altered enzymes with low activity. As a result, cells may burst due to the high level stress of ROS. Here molecular dynamics simulation of selected G6PD mutants in the dimer interface was carried out to understand the effect of mutation on protein stability and flexibility. RMSD was selected as primary criteria to determine structural stability. A higher value of RMSD indicated the compromised stability of protein. To find out the possible reason behind the change in stability, flexibility of protein was accessed by calculating RMSF from last frame of stable trajectories which showed variations as compared to wildtype. Secondary structure analysis was carried out to understand the changes in secondary structure elements.

Mutant A335T, known as G6PD Chatham, is the second most abundant G6PD mutation after G6PD Mediterranean which is associated with neonatal jaundice. RMSD of A335T was comparable to the wildtype. This could be attributed due to the nature of the wildtype amino acid versus that in mutated residue. *e.g*., in A335T, with alanine having the hydrophobic side chain replaced by threonine in A335T with the uncharged polar side chain which does not alter any side chain interactions with the protein, as shown in Figs. 3A–3B. The fluctuation in RMSD as compared to wildtype indicated structural perturbation which is also evident from the experimental studies showing that mutation of Alanine at position 335 with Threonine exhibits decreased affinity for the substrate *i.e.*, G6P (Vulliamy et al., 1988).The Threonine is polar amino acid with bigger uncharged side chain as compared to Alanine which has different affinity for substrate in wildtype. Also, Alanine in wildtype is the part of loop region at the end of extensive β-sheet network close to dimer interface (Fig. 3A). In order to accommodate large threonine there may be a change in the native contacts of protein resulting in the decrease of overall stability of protein.

Mutant R393H, also known as G6PD Nashville, lies in the region closer to the structural NADP^+^ binding site between the dimer interfaces. Comparisons of kinetics between wildtype and R393H indicated that k_m_ values for NADP^+^ and G6P were higher for R393H mutant as compared to wildtype G6PD (Syeda Rehana & Zaheer, 2018). The deleterious effect of the mutation could be plausible leading to lower binding capacity with coenzyme and lesser stability of protein as indicated by higher RMSD of mutant R393H when compared with wildtype. A decrease in native contacts has reduced the flexibility of binding site residues indicating that there exist three groups of interacting residues in dimer interface on βG, βK, βL lying closer in sequence but stretch from G6P binding site to structural NADP ^+^ binding site. It implied that residues Asn363 and Glu364 lying in the loop between βJ and βK interacted indirectly to 2′ phosphate group of structural NADP^+^ via bound water molecule. Residue next in sequence *i.e.*, Arg365 interacted to the O7 and O9 atoms of phosphate in G6P while neighboring Lys 366 developed direct contact to 2′ phosphate group of structural NADP^+^. In beta sheet; βG Lys 238 interacted indirectly with structural NADP^+^ whereas adjacent residue; Glu 239 got involved in hydrogen bond formation with O4 atom of G6P. Similarly Gln 395 of βL strand interacted with O atom of phosphate in G6P whereas Arg393 built interaction with nicotinamaide ring of structural NADP^+^ (Gómez-Manzo et al., 2014). The presence of substrate in the binding site is connected to structural NADP^+^ via these interacting residues. Mutation in these sites may have a disruptive effect on the G6P binding site as well resulting in overall decrease in structural flexibility (Gómez-Manzo et al., 2014). Experimental studies show that mutant R393H has more exposed hydrophobic areas on treatment with different concentrations of urea as compared to wildtype which indicated that mutation greatly reduces the activity of enzymes when compared to wildtype (Wang, Lam & Engel, 2006). Our RMSD and RMSF results showing decrease in structural stability and flexibility complemented the experimental findings of researcher however exact mechanism of dynamics was unknown previously which has been explored here providing information even at residual contact level.

Mutant V394L, known as G6PD Alhambra, has been classified as class I deficiency. The amino acid residue lies in the extensive β-sheet network in dimer interface in vicinity of structural NADP^+^. Residues in the β-sheets are known to form hydrogen bonds between the strands. The mutations in the β-sheet may be expected to obstruct normal hydrogen bond formation which was observed in V394L. Our study revealed that there is no direct contact of the residue with structural NADP^+^ however residues in the vicinity are in direct contact with the cofactor *i.e.*, Arginine 393 (Fig. 12) . Incorporation of larger Leucine in V394L as compared to smaller sized Valine in wildtype may have changed the local contacts and hence stability which is evident from higher RMSD and RMSF values. This local conformational change may affect the binding of structural NADP ^+^ disrupting the normal protein stability and function.

Our study is in line with the previously reported (Nguyen, Nguyen & Le, 2016) computational studies indicating that the mutation in the binding site of substrate and structural NADP^+^ result in the altered functioning of the enzyme by changing the alteration in the interaction of neighboring residues.

## Conclusions

The current study focuses on the dynamic properties of the G6PD wildtype and its three mutants to exploit their effect on the stability of the G6PD structure by MD simulations. Among these, A335T exhibited fewer fluctuations in these properties. Mutants R393H and V394L lying in the dimer interface and closer to structural NADP^+^, caused instability in protein structure. A decrease in stability may lead to a decrease in the catalytic activity of enzymes as a result of conformational modifications and reorganization which indicated that the changes in the region around the structural NADP^+^ had an effect on protein stability and hence activity. Alteration in this region severely affects the protein function leading to class-I deficiency.

Mutations in the dimer interface resulted in the change of interaction pattern of residues in the vicinity indicating the predominant role of dimer interface residue in maintaining the protein stability and flexibility. The protein stability is a key factor in determining the functionality of protein. The stability of protein can be affected by mutations in two ways: either by destabilizing or overstabilizing; consequently, deterioration of protein function by change of physico-chemical properties as a result of mutation. The binding free energy calculations performed by Molecular Mechanics Poisson Boltzmann Surface Area (MM-PBSA) quantified greater stability and spontaneity of wildtype complex with ligands as compared to A335T, R393H and V394L complexes with ligand. Free energy decomposition analysis revealed vdW energy as the major important factor facilitating the protein-ligand binding.

Naturally, a large number of G6PD mutants are found; therefore, the experimental observations of all mutants are not practical. However, the computational procedures can efficiently predict and support the experiments in evaluating a significant number of mutants. As no drug for G6PD is available to date, our study will provide insight into the structure-based drug designing to inhibit the effect of mutation and maintain the normal enzyme function. Also, exploiting a particular mutation can provide an insight to develop structure-based inhibitors against G6PD for cancer cells.

## Additional Information and Declarations

### Data Availability

The following information was supplied regarding data availability: The raw data is available in the Supplemental Figures, Table 1, and UniProt: rs137852316, rs137852335, and rs5030869.
